# Hypoimmune anti-CD19 chimeric antigen receptor T cells provide lasting tumor control in fully immunocompetent allogeneic humanized mice

**DOI:** 10.1038/s41467-023-37785-2

**Published:** 2023-04-10

**Authors:** Xiaomeng Hu, Karl Manner, Rowena DeJesus, Kathy White, Corie Gattis, Priscilla Ngo, Christopher Bandoro, Eleonore Tham, Elaine Y. Chu, Chi Young, Frank Wells, Ronald Basco, Annabelle Friera, Divy Kangeyan, Pascal Beauchesne, William E. Dowdle, Tobias Deuse, Terry J. Fry, Aaron E. Foster, Sonja Schrepfer

**Affiliations:** 1 Sana Biotechnology Inc., 1 Tower Place, South San Francisco, CA USA; 2 Present Address: Department of Surgery, Division of Cardiothoracic Surgery, Transplant and Stem Cell Immunobiology (TSI)-Lab, University of California San Francisco, San Francisco, CA USA

**Keywords:** Cancer, Humoral immunity

## Abstract

Manufacturing autologous chimeric antigen receptor (CAR) T cell therapeutics is complex, and many patients experience treatment delays or cannot be treated at all. Although current allogeneic CAR products have the potential to overcome manufacturing bottlenecks, they are subject to immune rejection and failure to persist in the host, and thus do not provide the same level of efficacy as their autologous counterparts. Here, we aimed to develop universal allogeneic CAR T cells that evade the immune system and produce a durable response. We generated human hypoimmune (HIP) T cells with disrupted *B2M, CIITA*, and *TRAC* genes using CRISPR-Cas9 editing. In addition, CD47 and anti-CD19 CAR were expressed using lentiviral transduction. These allogeneic HIP CD19 CAR T cells were compared to allogeneic CD19 CAR T cells that only expressed the anti-CD19 CAR (allo CAR T). In vitro assays for cancer killing and exhaustion revealed no differences between allo CAR T and HIP CAR T cells, confirming that the HIP edits did not negatively affect T cell performance. Clearance of CD19^+^ tumors by HIP CAR T cells in immunodeficient NSG mice was comparable to that of allo CAR T cells. In fully immunocompetent humanized mice, HIP CAR T cells significantly outperformed allo CAR T cells, showed improved persistence and expansion, and provided lasting cancer clearance. Furthermore, CD47-targeting safety strategies reliably and specifically eliminated HIP CAR T cells. These findings suggest that universal allogeneic HIP CAR T cell-based therapeutics might overcome the limitations associated with poor persistence of allogeneic CAR T cells and exert durable anti-tumor responses.

## Abstract (web summary)

The development of allogeneic chimaeric antigen receptor (CAR) T cells could overcome manufacturing bottlenecks for immunotherapy. However, immune rejection reduces the persistence and efficacy of these cells. Here, the authors generate allogeneic anti-CD19 CAR T cells that can evade the immune system and provide durable anti-tumour responses.

## Introduction

Autologous chimeric antigen receptor (CAR) T cells have successfully been used to treat hematological malignancies in many patients^1,2^. However, such custom-made cell products are expensive^3^, require complex patient-specific manufacturing with a failure rate of 2-10% in the commercial setting^4^, have limited scalability, and show large variability in quality between patients^5^ that can lead to unpredictable potency^6^. The delay in treatment from the manufacturing process can be particularly problematic in patients with rapidly progressing disease^7^. The development of allogeneic, ‘off-the-shelf’ CAR T cells may overcome some of these challenges, leading to increased access to such therapies. Allogeneic approaches face two major obstacles. First, the administered allogeneic CAR T cells may cause graft-versus-host disease (GVHD), and second, they may be rapidly eliminated by the host immune system (host-versus-graft disease, HVGD), limiting their anti-tumor activity^4,6^. Several approaches involving editing of immune-related genes are being investigated to prevent or alleviate these problems. Elimination of the endogenous T cell receptor (TCR) through the disruption of the *TRAC* locus has been demonstrated to prevent GVHD without affecting CAR-dependent effector functions^8^. To reduce the immunogenicity of T cells, we and other groups developed immune editing strategies to prevent rejection by the host immune system. Various approaches have entered early clinical trials^9^. Some allogeneic CAR T cell products still possess key immune recognition molecules, including MHC class I and II, requiring chemotherapeutic, biological, or both approaches to suppress host immunity to allow engraftment and persistence of allogeneic CAR T cells. One approach uses TCR- and CD52-depleted, but MHC class I- and II-replete CAR T cells, combined with the cytotoxic anti-CD52 antibody, alemtuzumab, to deplete host immune cells while sparing engineered allogeneic CAR T cells to enable engraftment and expansion^10^. However, prolonged treatment with anti-CD52 may increase toxicity (e.g., cytopenia) and risk of opportunistic infections^10^. Despite the use of host conditioning, the anti-tumor response and persistence of these engineered cell therapeutics may still be less effective than that of autologous CAR T treatment^4^. One way to prevent rejection includes the elimination of the human leukocyte antigens (HLA), which are the most important determinants of allorejection, through disruption of *B2M* and *CIITA*^11^. Since these edits prevent T cell-mediated allorejection, but render the cells susceptible to innate immune killing^12^, we additionally overexpress CD47 to protect against innate immune cell killing^13^. This hypoimmune (HIP) *B2M*^*−/−*^ *CIITA*^*−/−*^ CD47^+^ phenotype was previously shown to protect several cell types from all immune cell killing^14^.

The aim of this study was to generate HIP CAR T cells targeting CD19^+^ tumors. Here, we report that HIP CAR T cells are safe and achieve persistence and efficacy in fully allogeneic immunocompetent humanized mice. The advancement over existing strategies is that HIP CAR T cells avoid immune recognition and do not require the depletion of the host immune system to escape rejection. HIP editing might therefore improve the efficacy of allogeneic CAR T cells to levels so far only achieved with autologous products and increase timely access to cancer therapeutics for bigger patient populations.

## Results

### Engineering of HIP CAR T cells

**Fig. 1:**
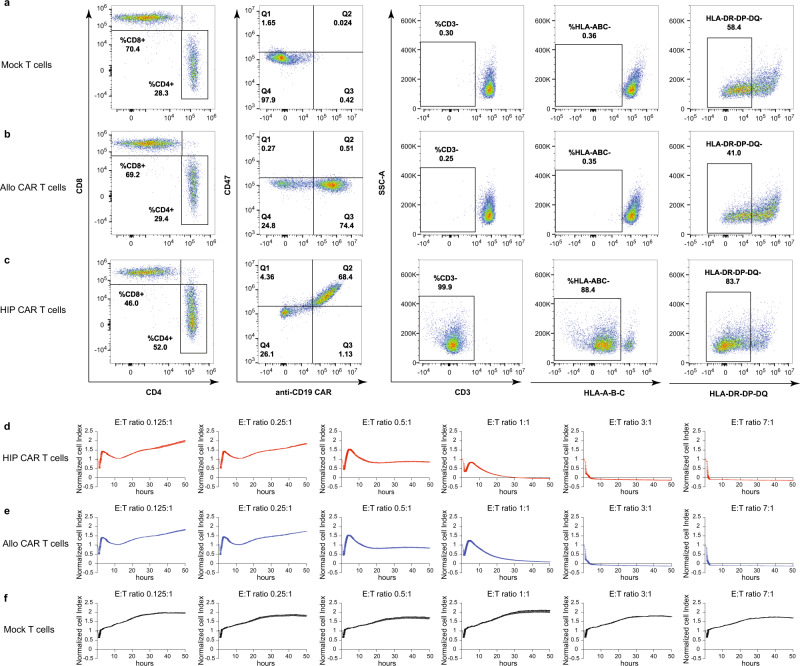
HIP CAR T cells are effective killers of Nalm6 tumor cells. **a**–**c** Flow cytometry of unedited (Mock, **a**) T cells, anti-CD19 allo CAR T cells (**b**), and anti-CD19 HIP CAR T cells (**c**) are shown. T cells are identified by CD4 or CD8 expression. Allo CAR T and HIP CAR T cells showed expression of the anti-CD19 CAR of approximately 70%. HIP CAR T cells showed disruption of the TCRαβ/CD3 complex and of HLA class I and II and overexpression of CD47 (representative graph of two independent experiments). **d**–**f** In vitro impedance cytotoxicity assay with Nalm6 target cells and HIP CAR T cells (**d**), allo CAR T cells (**e**), or Mock T cells (**f**) in different E:T ratios (mean ± SD, three independent replicates per group and time point).

T cells were isolated from leukopaks from healthy volunteers. Unedited T cells (Mock T cells) had a ~70:30 CD8^+^ to CD4^+^ T cell ratio, with uniform CD3 expression, were all HLA class I-positive, and about half of the population was HLA class II-positive (Supplementary Fig. 1a; Fig. 1a–c). HIP CAR T cells were engineered as follows. T cells underwent CRISPR-Cas9 editing of their *B2M* and *CIITA* genes to disrupt HLA class I and II expression. In addition, the *TRAC* locus was also edited using CRISPR-Cas9 to prevent GVHD. Disruption of *TRAC* not only prevents expression of TCRα, but also disrupts cell-surface CD3ε expression and assembly of the multimeric TCRαβ/CD3 complex. CD47 and anti-CD19 CAR overexpression was achieved using transduction with a bicistronic lentiviral vector encoding anti-CD19 CAR and CD47. The engineered T cells were then magnetically enriched for CD3 negative T cells. The HIP CAR T bulk population was 68.4% positive for CD47 and anti-CD19 CAR, and 88.4% and 83.7% negative for HLA class I or II, respectively. Allogeneic CAR T control cells (allo CAR T) were generated using a bicistronic lentiviral vector encoding anti-CD19 CAR and truncated epidermal growth factor receptor (EGFRt)^15^. Approximately 75% of the allo CAR T cells were positive for the anti-CD19 CAR.

To assess whether the HIP engineering together with the *TRAC* knockout affect the killing capacity of CAR T cells, we performed in vitro impedance cytotoxicity assays with CD19^+^ Nalm6 target cells (Fig. 1d–f). HIP CAR T cells, allo CAR T cells and Mock T cells were used as effector cells in a range of effector-to-target cell (E:T) ratios. CD19^+^ Nalm6 cancer cells were killed to a similar extent by HIP CAR T cells and allo CAR T cells, showing that HIP edits do not interfere with target recognition by the CAR or T cell function. Mock T cells showed no cytotoxicity against Nalm6 target cells.

### HIP CAR T cells are effective at clearing tumors in vivo

**Fig. 2:**
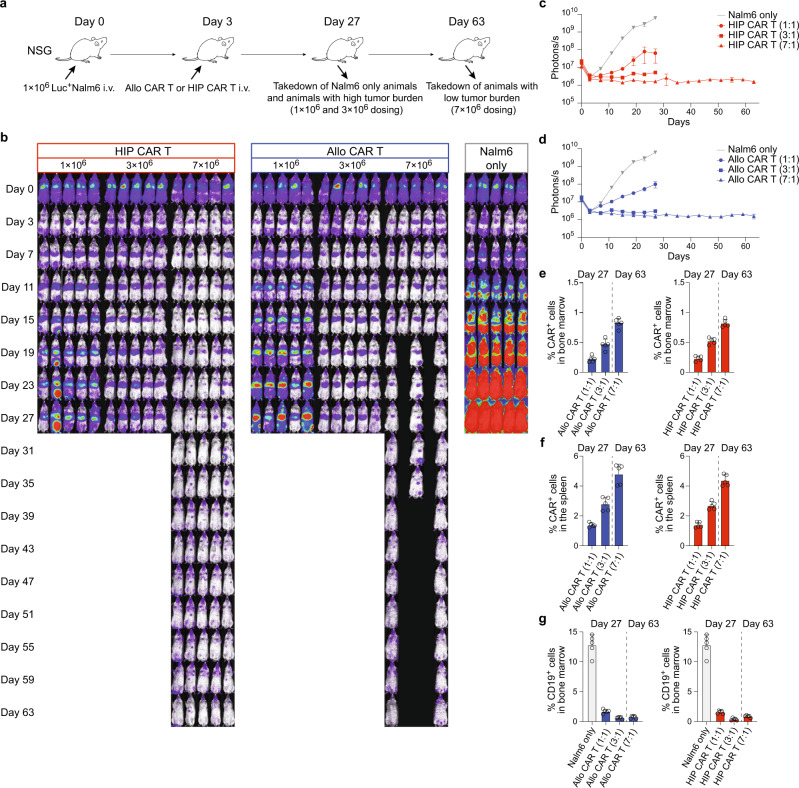
Nalm6 tumor killing in NSG mice. **a** Immunodeficient NSG mice were injected with 1 × 10^6^ Luc^+^ Nalm6 cells via the tail vein and were followed by BLI. Some mice then received allo CAR T cells or HIP CAR T cells (*n* = 5 mice in each group in one experiment) intravenously on day 3. Spleen and bone marrow were taken after 27 or 63 days. **b** BLI images show the tumor burden for all mice in this study. **c**, **d** Graphs show BLI signals for animals in the HIP CAR T (**c**) and allo CAR T cell groups (**d**, mean ± SEM, *n* = 5 animals per group). **e**, **f** The percentage of CAR^+^ cells in the bone marrow (**e**) and spleen (**f**) were assessed on the day of organ recovery (mean ± SD, *n* = 5 animals per group). There were no significant differences between the two CAR T cell groups at the same dose level. **g** The percentage of CD19^+^ cells in the bone marrow was assessed on the day of recovery (mean ± SD, *n* = 5 animals per group). For (**e**–**g**): Unpaired, two-tailed Student’s *t* test was used to compare the allo and HIP CAR T cell groups at the same dose level, and no differences were found.

The efficacy of HIP CAR T cells to clear Nalm6 tumors was assessed in immunodeficient NSG mice. Mice were injected with one million Nalm6 cells into their tail veins (Fig. 2a). The tumor cells were transduced to express firefly luciferase (Luc), so the cancer burden could be assessed quantitatively throughout the study using bioluminescence imaging (BLI, Fig. 2b). Three days later, they received 1, 3, or 7 million allo CAR T cells or HIP CAR T cells intravenously. Both CAR T cell products were dosed based on CAR^+^ cells. Bone marrow and spleen were recovered after 27 days in the groups receiving Nalm6 only and those with the lower doses of 1 or 3 million CAR T cells. The two high-dose groups were continued until day 63. Nalm6 cancers grew quickly, and their progression was similarly suppressed by allo CAR T and HIP CAR T cells in a dose-dependent manner (Fig. 2c, d). However, 3 of 5 mice in the allo CAR T high-dose group were euthanized due to signs of GVHD (skin score of 1.5 and loss of body weight). The amount of CAR^+^ cells in bone marrow and spleen was highest with the 7 million doses (Fig. 2e, f; Supplementary Fig. 2a, b). The cancer burden in the bone marrow was similarly reduced in the allo CAR T and HIP CAR T cell groups (Fig. 2g; Supplementary Fig. 2c). The study was then repeated with the CD19^+^ Burkitt’s lymphoma cell line, Daudi (Supplementary Figs. 3,  4). We again observed a dose-dependent effect on tumor clearance for both allo CAR T and HIP CAR T. However, with this tumor cell line, only HIP CAR T cells in their highest dose of 7 million cells were able to completely suppress tumor growth and thus outperformed allo CAR T cells. One animal in the allo CAR T group died due to high tumor burden; and 2 animals in the HIP CAR T groups were found dead after imaging. The content of CAR^+^ cells in the bone marrow and spleen was higher and the tumor burden lower with HIP CAR T cells. Overall, HIP CAR T cells showed at least equal efficacy for tumor killing in immunodeficient models.

### HIP CAR T cells are not prone to exhaustion

**Fig. 3:**
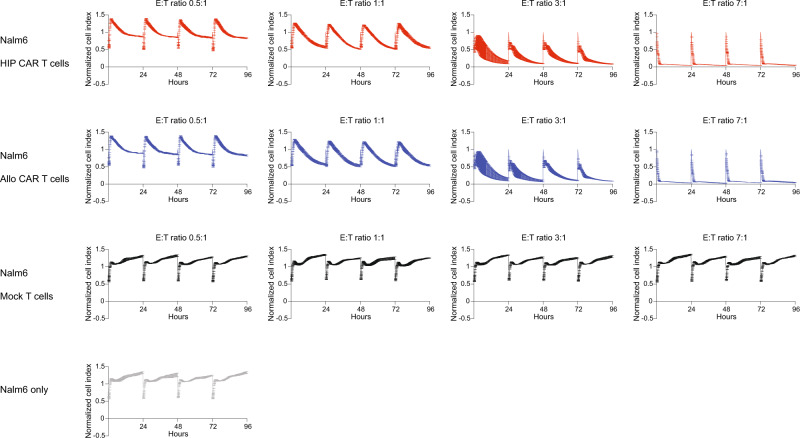
CAR T cells are repeatedly re-challenged with Nalm6 tumor cells in vitro. Different doses of HIP CAR T cells, allo CAR T cells, or Mock T cells were used as effectors against Nalm6 target cells in impedance cytotoxicity assays (mean ± SD, three independent replicates per group and time point in one experiment). The T cells were recovered daily and used as effector cells in new wells with Nalm6 target cells for 4 days. There was no difference in cytotoxicity between the two CAR groups at the same dose level.

**Fig. 4:**
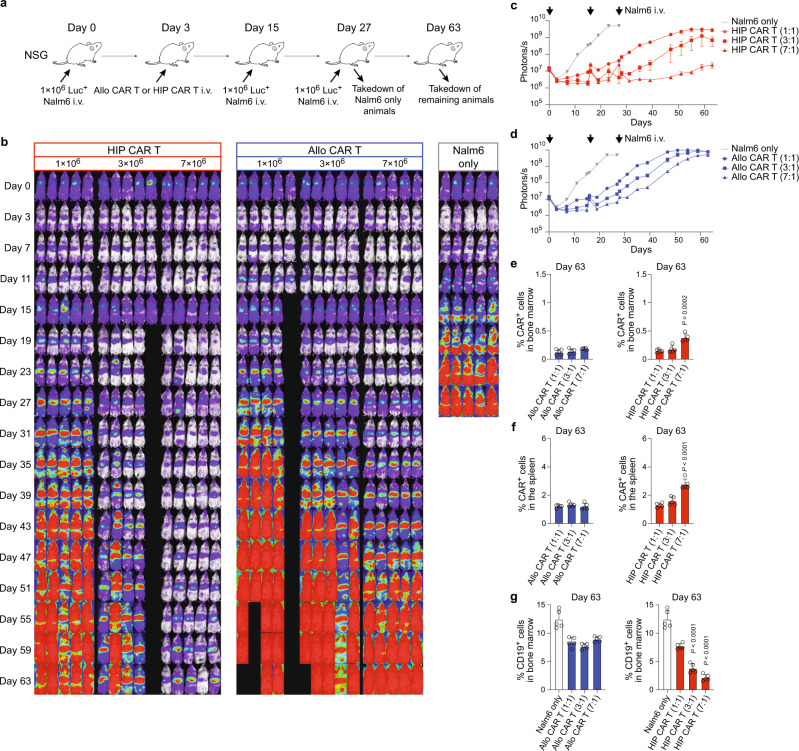
CAR T cells are repeatedly re-challenged with Nalm6 tumor cells in vivo. **a** Immunodeficient NSG mice were injected with 1 × 10^6^ Luc^+^ Nalm6 cells and received different doses of allo CAR T cells or HIP CAR T cells on day 3. The mice were injected again on days 15 and 27 with Nalm6 cells. Spleen and bone marrow was taken after 27 or 63 days. **b** BLI images show the tumor burden for all mice in this study. **c**, **d** Graphs show BLI signals for animals in the HIP CAR T (**c**) and allo CAR T cell groups (**d**, mean ± SEM, *n* = 5 animals per group in one experiment, arrows indicate Nalm6 injections). **e**, **f** The percentage of CAR^+^ cells in the bone marrow (**e**) and spleen (**f**) were assessed on the day of organ recovery (mean ± SD, *n* = 5 animals per group). Significant differences between the two CAR T cell groups at the same dose level (7:1 ratio) are shown. **g** The percentage of CD19^+^ cells in the bone marrow was assessed on the day of recovery (mean ± SD, *n* = 5 animals per group). For (**e**–**g**): Unpaired, two-tailed Student’s *t* test was used to compare the allo and HIP CAR T cell groups at the same dose level and significant differences are shown.

Given their high efficacy in vitro and in vivo, we next assessed whether HIP CAR T cells are more susceptible to exhaustion. In vitro impedance cytotoxicity assays were performed with Nalm6 target cells and a range of allo CAR T and HIP CAR T cell E:T ratios. Every 24 h for four days, the effector cells were recovered from the electrode plate wells and were added to new wells with fresh Nalm6 cells (Fig. 3). Allo CAR T and HIP CAR T cells showed dose-dependent Nalm6 killing efficacy, but there was no reduction of cytotoxicity over time or with repeated cancer exposures in vitro. For another in vitro assay, phenotypic exhaustion markers on allo CAR T and HIP CAR T cells were assessed by flow cytometry before (Supplementary Figs. 1b,  5a, b) or after (Supplementary Fig. 5c, d) incubation with Nalm6 cancer cells for 90 h. Both CAR T populations did not express TIM3, TIGIT, LAG3, CD39, or CTLA-4 and no upregulation was induced through CAR stimulation during the time of the assay. PD-1 was expressed in both CAR T populations, but we did not observe changes after Nalm6 incubation. Also, allo CAR T and HIP CAR T cells did not change their IFN-γ or IL-2 secretion capacity between 24 and 90 h of Nalm6 co-culture (Supplementary Fig. 5e, f). To confirm stable cancer killing efficacy in vivo, NSG mice were injected with one million Nalm6 cells and were re-injected twice after 15 and 27 days (Fig. 4a). Different doses of allo CAR T or HIP CAR T cells injected on day 3 showed dose-dependent efficacies (Fig. 4b–d). Three animals in the allo CAR T low-dose group and one animal in the allo CAR T high-dose group died of high tumor burden and tumor re-dosing complication, respectively. One animal in the HIP CAR T medium-dose group died due to body weight loss for unidentified reason. In the 7 million (high-dose) groups, the HIP CAR T cells were more effective in controlling repeated Nalm6 cancer cell injections than allo CAR T cells and kept the tumor burden at significantly lower levels throughout the study period. On day 63, HIP CAR T cells showed significantly higher CAR^+^ cell numbers in the bone marrow and spleen and more effectively reduced CD19^+^ cancer cells in the bone marrow (Fig. 4e–g; Supplementary Fig. 6). The expression of most exhaustion markers on both allo CAR T cells and HIP CAR T cells recovered from the spleens were low, with the exemption of PD-1, which had been highly expressed even before the injection into mice (Supplementary Figs. 1d,  7,  8). We did not see differences in CAR T cell exhaustion between groups. Together, these data show lasting anti-tumor efficacy of HIP CAR T cells in these models.

### HIP CAR T cells can effectively be cleared with CD47-targeting strategies

**Fig. 5:**
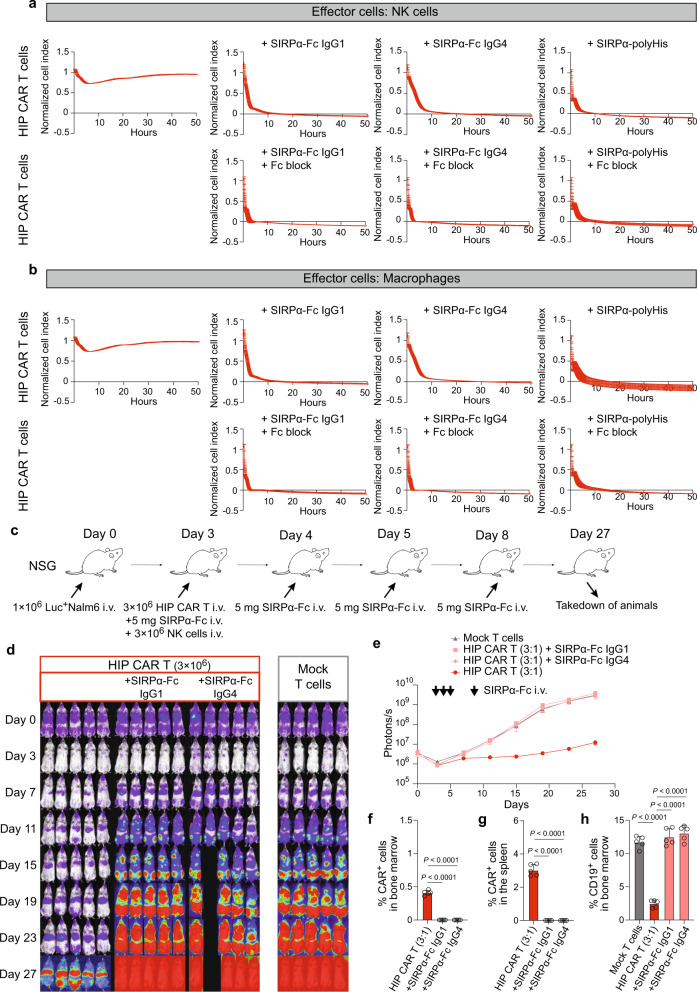
The CD47-targeting safety strategy for HIP CAR T cells. **a**, **b** HIP CAR T cells were challenged with NK cells (**a**) or macrophages (**b**) in the presence and absence of SIRPα-Fc IgG1 or IgG4, or a SIRPα with poly-His tail (upper rows, three independent replicates per group and time point). Effector cell Fc was blocked by Fc block solution (lower rows, three independent replicates per group and time point in one experiment). **c** Immunodeficient NSG mice were injected with 1 × 10^6^ Luc^+^ Nalm6 cells and received 3 × 10^6^ HIP CAR T cells or Mock T cells on day 3. In two HIP CAR T groups, mice additionally received three doses of the SIRPα-Fc IgG1 or IgG4 on days 3, 4, 5, and 8. Spleen and bone marrow were taken after 27 days. **d** BLI images show the tumor burden for all mice in this study. **e** Graphs show BLI signals for animals receiving HIP CAR T cell with or without the SIRPα-Fc IgG1 or IgG4 (mean ± SEM, *n* = 5 animals per group in one experiment, arrows indicate SIRPα-Fc i.v. injections). **f**, **g** The percentage of CAR^+^ cells in the bone marrow (**f**) and spleen (**g**) were assessed on day 27 (mean ± SD, *n* = 5 animals per group). No HIP CAR T cells were detected in animals that received either fusion protein (ANOVA with Bonferroni’s post hoc test). **h** The percentage of CD19^+^ cells in the bone marrow was assessed on day 27 (mean ± SD, *n* = 5 animals per group). There were no differences between the animals receiving Mock T cells or HIP CAR T cells with either fusion protein (ANOVA with Bonferroni’s post hoc test).

HIP CAR T cells are a new class of allogeneic immunotherapeutics with the potential to escape adaptive and innate host immune responses. We therefore tested a safety strategy to eliminate these cells in case their persistence would cause harm to the patient. Since HIP CAR T cells have increased CD47 expression, we hypothesized delivery of CD47-targeting fusion proteins would interfere with CD47 functionality and deplete HIP CAR T cells by enabling host innate cell killing. We first tested SIRPα-Fc with human IgG1 or IgG4 Fc in vitro. HIP CAR T cells were used as targets and human NK cells (Fig. 5a) or macrophages (Fig. 5b) as effector cells. With both the IgG1 and IgG4 fusion proteins, the HIP CAR T cells were killed quickly by NK cell and macrophage effector cells. The killing kinetics remained unaffected by Fc block and thus suggests that the fusion proteins counteract the protective effect of CD47 on innate immune cells, but the killing seems not Fc-dependent. Furthermore, a SIRPα fusion protein with a poly-His tail lacking any Fc was similarly effective as the IgG1 and IgG4 Fc fusion proteins. Next, the clearance of HIP CAR T cells was tested in vivo. NSG mice with adoptive transfer of human NK cells were injected with one million Nalm6 cells and received three million HIP CAR T cells three days later. The SIRPα-Fc fusion proteins were given intravenously four times on days 3, 4, 5, and 8 at 5 mg per dose (Fig. 5c). Mock T cells without CD19 CAR were used as controls (Fig. 5d). Both SIRPα-Fc fusion proteins effectively abolished the anti-tumor efficacy of HIP CAR T cells as assessed by BLI (Fig. 5e). One animal in the SIRPα-Fc IgG4 group was found dead after imaging. No CAR^+^ cells could be detected in the bone marrow and spleen at the end of the study (Fig. 5f, g; Supplementary Fig. 9a, b). The tumor burden of CD19^+^ cells in bone marrow remained unaffected by the HIP CAR T cells when either of the fusion proteins were used (Fig. 5h; Supplementary Fig. 9c). We next quantified CD47 expression on a variety of different benign tissue types to assess the risk for off-target cytotoxicity from the use of the SIRPα-Fc IgG1 or IgG4 fusion proteins. CD47 was ubiquitously expressed on all cell types tested, although the expression was lower when compared to HIP CAR T cells (Supplementary Figs. 1c,  10a–b,  11). Cytotoxicity assays against primary human fibroblasts, mesenchymal stromal cells, endothelial cells, T cells, and allo CAR T cells were performed with the SIRPα-Fc and poly-His fusion proteins and NK cells or macrophages as effector cells (Supplementary Figs. 10c, d,  12). We did not observe any killing with any fusion protein or effector cell in any cell type tested. Thus, a CD47-targeting safety strategy was effective and specific for the elimination of HIP CAR T cells and abolished their activity.

### The adaptive immunogenicity of subpopulations in the HIP CAR T cell bulk depends on the expression of HLA

**Fig. 6:**
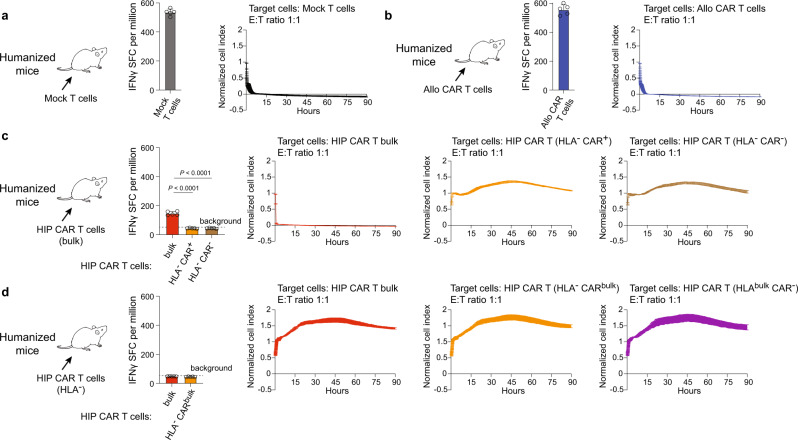
Immune response in humanized mice. Humanized mice were immunized with Mock T or CAR T cells. After 7 days, recipient T cells were isolated from spleen and IFNγ Elispot assays (Elispot: mean ± SD, *n* = 5 animals per group in one experiment, spot-forming cells (SFC) per million responder cells) and Xcelligence cytotoxicity assays (mean ± SD, three independent replicates per group and time point) were performed against the specified Mock T or CAR T cell population at an E:T ratio of 1:1. **a** Humanized mice that received allogeneic Mock T cells built a strong immune response against these cells, reflected by high Elispot frequencies and rapid target cell killing. **b** Similarly, humanized mice that received allogeneic CAR T cells built a strong immune response against these cells with high Elispot frequencies and rapid target cell killing. **c** Humanized mice that received allogeneic HIP CAR T bulk cells built a weak immune response against bulk cells, but no response against HLA^−^ CAR^+^ or HLA^−^ CAR^−^ cells. Accordingly, only bulk underwent killing, whereas and HLA^−^ CAR^+^ and HLA^−^ CAR^−^ cells survived (ANOVA with Bonferroni’s post hoc test). **d** Humanized mice that received the sorted HLA^−^ subpopulation of the HIP CAR T bulk did not build any response against HIP CAR T bulk, HIP CAR T (HLA^−^ CAR^bulk^) and HIP CAR T (HLA^bulk^ CAR^−^) cells and exerted no cytotoxicity.

The potential to avoid clearance of the HIP CAR T cells by the host immune system was studied next. NSG mice were reconstituted with human CD34^+^ cord blood-derived hematopoietic stem cells (HSC), and all humanized mice selected for this study showed at least 45% human CD45^+^ cells. Humanized mice received allo CAR T, HIP CAR T, or Mock T cells and after 7 days, T cells recovered from spleen were used as effector cells in ELISpot assays and impedance cytotoxicity assays. Using different target cell subpopulations, this setup allows the determination of the specificities of the host immune response. Mice that received Mock T cells generated immunity against Mock T cells and those were rapidly killed in vitro (Fig. 6a). Similarly, mice that received allo CAR T cells stimulated immune responses against allo CAR T cells (Fig. 6b). After receiving bulk HIP CAR T cells, T cells of humanized mice were still able to mount a small IFNγ response against bulk HIP CAR T cells. HLA^−^ CAR^+^ and HLA^−^ CAR^−^ T cells, however, did not induce an immune response and were not killed in vitro (Fig. 6c). We thus hypothesized allogeneic HLA^+^ cells in the HIP CAR T cell bulk would prime the humanized mice against HLA and subpopulations expressing HLA would still be rejected. We therefore sorted HLA^−^ cells from the HIP CAR T cell bulk and injected them into humanized mice. T cells recovered from these mice did not induce any immune response against all HIP CAR T bulk cells, including HLA^−^ CAR^bulk^ and HLA^bulk^ CAR^−^ subpopulations (Fig. 6d). This confirmed that HLA-expressing cells in the HIP CAR T cell bulk were still able to immunize the host and induce an HLA-directed response in these models.

### HIP CAR T cells provide lasting tumor killing efficacy in fully immunocompetent allogeneic humanized mice

**Fig. 7:**
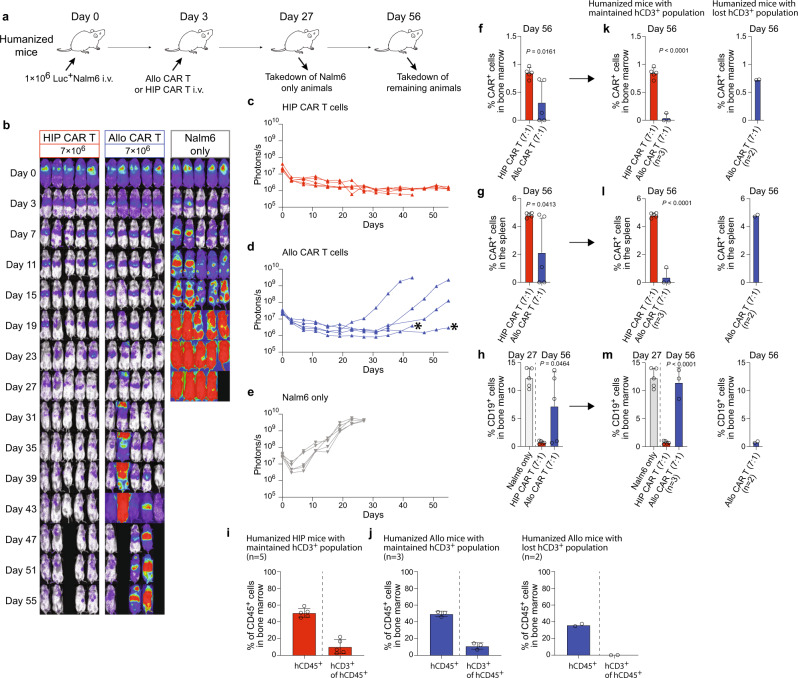
HIP CAR T cells provide lasting tumor control in fully immunocompetent allogeneic humanized mice. **a** Humanized mice were injected with 1 × 10^6^ Luc^+^ Nalm6 cells and received 7 × 10^6^ allo CAR T cells or HIP CAR T cells on day 3 (one experiment). Spleen and bone marrow were taken after 27 or 56 days. **b** BLI images show the tumor burden for all mice in this study. **c**–**e** Graphs show BLI signals for animals in the HIP CAR T (**c**) and allo CAR T cell groups (**d**), and the Nalm6 only group (**e**, all single animals are shown). * indicates outliers that got assessed further. **f**, **g** The percentages of CAR^+^ cells in the bone marrow (**f**) and spleen (**g**) were assessed on day 56 (mean ± SD, *n* = 5 animals per group). Significant differences between the two CAR T cell groups are shown. **h** The percentage of CD19^+^ cells in the bone marrow was assessed on day 56 as endpoint (mean ± SD, *n* = 5 animals per group) and were significantly different. Endpoint values from earlier euthanized mice in the allo CAR T group due to high tumor burden were included in the day 56 graphs. **i**, **j** Reconstitution of human immune cells was assessed in the HIP CAR T (**i**) and the allo CAR T (**j**) groups after euthanasia. The percentage of hCD45^+^ cells among all CD45^+^ cells and the percentage of hCD3^+^ cells in the hCD45^+^ population was quantified (mean ± SD, *n* = 5 HIP animals, *n* = 3 allo animals with maintained hCD3^+^ population and *n* = 2 allo animals with lost hCD3^+^ population). **k**, **l** The percentages of CAR^+^ cells in the bone marrow (**k**) and spleen (**l**) were assessed separately for animals that had maintained their hCD3^+^ population (mean ± SD, *n* = 5 in the HIP CAR T group and *n* = 3 in the allo CAR T group). The 2 animals in the allo CAR T group that had lost their hCD3 population are shown separately. **m** The percentage of CD19^+^ cells in the bone marrow was assessed separately for animals that had maintained their hCD3^+^ population (*n* = 5 in the HIP CAR T group and *n* = 3 in the allo CAR T group, mean ± SD). The 2 animals in the allo CAR T group that had lost their hCD3 population are shown separately. For (**f**–**h**) and (**k**–**m**): Unpaired, two-tailed Student’s *t* test was used to compare HIP and allo CAR T cell groups and significant differences are shown.

The Nalm6 killing efficacy was next assessed in immunocompetent humanized mice. In this model, the allogeneic immunotherapeutic cells are exposed to the host immune system, which can jeopardize their persistence. Humanized mice were injected with one million Nalm6 cells and received 7 million allo CAR T or HIP CAR T cells on day 3 (Fig. 7a). Longitudinal BLI showed that HIP CAR T cells provided lasting tumor suppression throughout the study period (Fig. 7b, c). Allo CAR T cells slowed the progression of tumor growth only temporarily but failed to persist and therefore failed to control cancer progression (Fig. 7d, e). One mouse in the Nalm6 and allo CAR T groups were euthanized due to high tumor burden. One mouse in the allo CAR T and HIP CAR T was found dead with BC2 score. On average, HIP CAR T cells showed significantly higher numbers of CAR^+^ cells in the bone marrow and spleen and significantly lower CD19^+^ tumor burden in the bone marrow (Fig. 7f, h). However, we observed two outliers in the allo CAR T group in which Nalm6 cancers were well controlled (* in Fig. 7d). We therefore quantified the reconstitution of human immune cells again at the end of the observation period. All mice in the HIP CAR T group (Fig. 7i; Supplementary Fig. 13a), but only 3 mice in the allo CAR T group (Fig. 7j; Supplementary Fig. 13b) still met our criteria of >2% hCD3^+^ cells of the hCD45^+^ population. The two animals in which the cancer was controlled both had lost their hCD3^+^ immune cell population. We therefore re-analyzed CAR T cell persistence and CD19^+^ tumor burden in immunocompetent animals. Allo CAR T cells were effectively rejected and the tumor burden in the bone marrow in the allo CAR T group was similar to that in the group that did not receive any CAR T product (Fig. 7k–m; Supplementary Fig. 13c–e). Interestingly, allo CAR T cells persisted and suppressed cancer growth in the two immune-incompetent animals. Thus, only HIP CAR T cells showed persistent efficacy in fully immunocompetent allogeneic hosts.

### HIP CAR T cells effectively prevent cancer growth in fully immunocompetent allogeneic humanized mice receiving Nalm6 re-injection

**Fig. 8:**
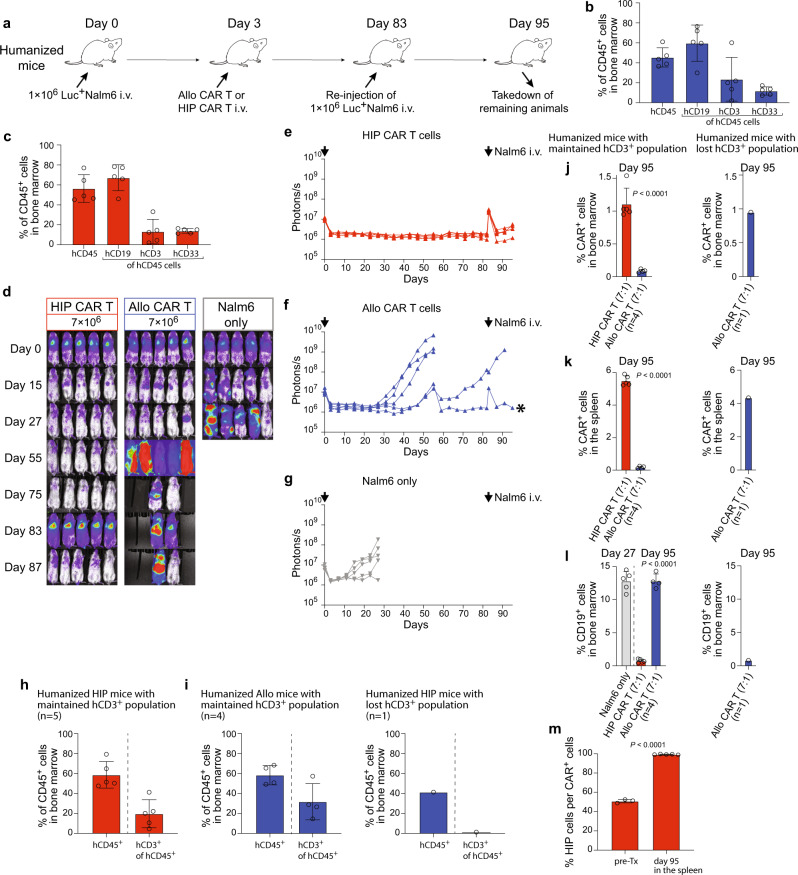
HIP CAR T cells quickly clear tumor cells re-injected after 83 days in fully immunocompetent allogeneic humanized mice. **a** Humanized mice were injected with 1 × 10^6^ Luc^+^ Nalm6 cells and received 7 × 10^6^ allo CAR T cells or HIP CAR T cells on day 3 (one experiment). Then, they received another injection of 1 × 10^6^ Luc^+^ Nalm6 cells on day 83. Spleen and bone marrow were taken after 95 days. **b**, **c** Human immune cell reconstitution is shown for all 5 mice in the allo CAR T group (**b**) and the HIP CAR T group (**c**). Individual values are shown and mean ± SD. **d** BLI images show the tumor burden for all mice in this study. **e**–**g** Graphs show BLI signals for animals in the HIP CAR T (**e**) and allo CAR T cell groups (**f**), and the Nalm6 only group (**g**, all single animals are shown). Arrows indicate Nalm6 injections, * indicates outliers used for further analysis. **h**, **i** Reconstitution of human immune cells was assessed in the HIP CAR T (**h**) and the allo CAR T (**i**) groups after euthanasia. The percentage of hCD45^+^ cells among all CD45^+^ cells and the percentage of hCD3^+^ cells in the hCD45^+^ population was quantified (mean ± SD, *n* = 5 HIP animals, *n* = 4 allo animals with maintained hCD3^+^ population and *n* = 1 allo animal with lost hCD3^+^ population). **j**, **k** The percentages of CAR^+^ cells in the bone marrow (**j**) and spleen (**k**) were assessed separately for animals that had maintained their hCD3^+^ population (*n* = 5 in the HIP CAR T group and *n* = 4 in the allo CAR T group, mean ± SD). The one animal in the allo CAR T group that had lost their hCD3 population is shown separately. Significant differences between the two CAR T cell groups are shown. **l** The percentage of CD19^+^ cells in the bone marrow was assessed separately for animals that had maintained their hCD3^+^ population (*n* = 5 in the HIP CAR T group and *n* = 4 in the allo CAR T group, mean ± SD). The one animal in the allo CAR T group that had lost their hCD3 population is shown separately. **m** The percentages of the HIP-edited cells in the HIP CAR T cell product before injection (*n* = 3) and the percentages of HIP-edited cells among the recovered CAR^+^ cell population from the spleen on day 95 (*n* = 5) are shown (mean ± SD, unpaired, two-tailed Student’s *t* test). For (**h**–**l**): Endpoint values from earlier euthanized mice in the allo CAR T group due to high tumor burden were included in the day 95 graphs to show all values at the individual endpoint. For (**j**–**l**): Unpaired, two-tailed Student’s *t* test was used to compare HIP and allo CAR T cell groups and significant differences are shown.

Humanized mice were injected with one million Nalm6 cells and received 7 million allo CAR T or HIP CAR T cells on day 3 (Fig. 8a). On day 63, they received a re-injection of another million Nalm6 cells to challenge the CAR T efficacy. All animals receiving HIP CAR T or allo CAR T cells met inclusion criteria for human immune cell reconstitution (Fig. 8b, c; Supplementary Fig. 14a, b). BLI follow-up showed that HIP CAR T cells provided lasting tumor suppression even when Nalm6 was re-injected (Fig. 8d, e). The minimal and very short-lived spike in BLI signal after the injection showed how effective and almost immediate the HIP CAR T cells cleared the additional tumor burden. Only two animals were still alive to receive the Nalm6 re-injection in the allo CAR T and one mouse showed quick subsequent cancer growth (Fig. 8f, g). Nalm6 remained controlled in one animal in the allo CAR T group (* in Fig. 8f). We re-analyzed human immune cell reconstitution at the end of the study period and found that the animal in the allo CAR T cell group that showed persistent tumor suppression had lost the hCD3^+^ immune cell population. All other animals still met inclusion criteria (Fig. 8h, i; Supplementary Fig. 14c, d). Among immunocompetent animals, we found HIP CAR T cell ratios in the bone marrow and spleen on day 95 that were very similar to those previously observed on day 56 (Fig. 8j, k; Supplementary Fig. 14e, f). CD19 tumors were effectively suppressed in the HIP CAR T group, while the tumor burden in the allo CAR T group was similar to the group not receiving any CAR T product (Fig. 8l; Supplementary Fig. 14g). Interestingly, while only approximately 50% of the cells in the injected HIP CAR T cell product showed the HIP phenotype, we could exclusively detect HIP CAR T cells in the spleen after 95 days (Fig. 8m; Supplementary Fig. 14h). All partially edited or unedited cells had vanished. This selection process underlines the necessity of the HIP phenotype for long-term persistence. The HIP CAR T cells provided effective cancer control in fully allogeneic immunocompetent humanized mice even after tumor re-injection.

## Discussion

Autologous CAR T cell products for hematological cancers have had an impressive impact on patients who had previously failed other conventional therapy approaches^16^. The initial success with CD19^+^ cancers has shown the potential of engineered immune oncology cell therapeutics and there is hope that new immune cell products will be successful in treating other types of cancer as well^17–19^. However, even for hematological malignancies, such as diffuse large B-cell lymphoma or multiple myeloma for which there are approved autologous CAR T products, supply currently falls short of the demand, demonstrating the unmet medical need for patients who cannot access autologous CAR T therapies^20^.

The ability to make large quantities of universal, allogeneic hypoimmune CD19 CAR T cell products could improve CAR T cell availability for more patients, which may have a dramatic impact on patient outcomes^21^. The HIP strategy utilized in this work has demonstrated protection of different cell types against all adaptive and innate immune cells^13,14,22^. This study demonstrates that HIP CAR T cells, which also include a disruption of the TRAC locus to prevent GVHD, show similar or superior tumor killing efficacy to allo CAR T cells. Using both in vitro assays and tumor studies in immunodeficient mice, we show that HIP engineering does not impact CAR T cell specificity, impair cytotoxic function, accelerate T cell exhaustion, or weaken anti-tumor efficacy against CD19^+^ Nalm6 and Daudi cancer cells. In immunocompetent models, HIP CAR T cells outperformed allo CAR T cells in tumor control, because all allo CAR T cells were rejected. HIP T effector cells reliably escaped both adaptive and innate immune cell killing and achieved persistence. Immunogenicity against allo CAR T cells may derive from alloantigens or transgenic proteins, including murine binders in the CAR (e.g., anti-CD19 FMC63), that produce both T cell and antibody-mediated immune responses^23^. Our data show that HIP CAR T cells are resistant to both adaptive immune responses and innate immune cell killing from both macrophage and NK cells. This suggests that despite HLA disparities between the allogeneic donor and the patient, or immunogenicity of the transgenic modifications, HIP cells are protected from host immune responses, preserving their persistence and anti-tumor activity. In humanized mice, which most comprehensively model the allogeneic patient setting, HIP CAR T cells were more effective than allo CAR T cells in eliminating CD19 + tumors and demonstrated prolonged CAR T cell persistence. Even when the Nalm6 cancer was re-injected after 12 weeks, the HIP CAR T cells very rapidly cleared the additional tumor burden. This not only speaks to the persistence of the HIP CAR T product, but also to its maintained tumor killing efficacy.

Due to their biological activity, therapies using CAR T cells can result in severe toxicities, including cytokine release syndrome (CRS) and immune effector cell-associated neurotoxicity syndrome (ICANS)^24^. Safety switches, including the co-expression of inducible Caspase 9 with a targeted CAR, have been shown to ameliorate CAR T-related toxicities when they occur^25^. Given the reliance on CD47 overexpression to protect MHC class I and II-disrupted CAR T cells against host innate immune cells, we evaluated whether the use of CD47-directed antibodies could “block” the protective mechanism of the CD47 overexpression to enable innate immune cell killing and serve as a safety switch to eliminate HIP CAR T cells. Here, we observed near complete ablation of HIP CAR T cell function using CD47-targeting fusion proteins. Furthermore, this strategy is highly specific and does not target other patient tissue cell types that express HLA. The Fc-independent kill mechanism by the SIRPα fusion proteins selectively blocks the protective CD47 effect and makes HLA-deficient HIP CAR T cells susceptible to rapid innate immune clearance. Our data support the efficacy of anti-CD47 approaches as a safety strategy to control potential HIP CAR T cell-related toxicities in future clinical use.

In summary, these data demonstrate that our HIP CAR T cells are effective immune therapeutics against CD19^+^ tumors, have a reduced risk of GVHD, and have the ability to escape both adaptive and innate immunity for improved persistence in allogeneic recipients. Because of this immune evasion feature, HIP CAR T cells have the potential to provide similar CAR T activity to that of autologous CAR T therapies, without the need for aggressive and more prolonged host immune suppression that may increase toxicity and risk of infections. In addition, we present a potential safety strategy with CD47-targeting fusion proteins for the selective elimination of the administered HIP CAR T cells. Together, these data support the potential use of allogeneic, universal off-the-shelf HIP CAR T cell products for CD19 + cancer therapy.

## Methods

### Production of lentiviral vectors

LV Max HEK 293 F cells (ThermoFisher, cat.no A35347) were grown in 200 mL suspension cultures and transfected at a cell density of 2.5 × 10^6^ cells/ml. The cells were transfected using PEI complexes (PEI:DNA of 2.5) containing VSV-G (34.56 μg), GagPol (45.36 μg), Rev (23.76 μg) and transfer vectors (112.32 μg) harboring CD19CAR-EGFRt, CD19CAR-CD47 or CD47-CD19CAR respectively. Cell cultures were harvested and clarified 2 days post transfection, followed by centrifugation for 5 h at 4000 g for concentration. Lentiviral pellets were resuspended to a final 250x concentrate. Lentiviral vector titers (TU/mL) were determined by serial dilution of vectors on primary T cells and the percentage of CAR-positive cells was measured by flow cytometry 7 days post-transduction.

### Gene editing of T cells

Human T cells were purchased from STEMCELL Technologies (Seattle, WA) or HemaCare Corporation (Northridge, CA). Human blood samples were collected utilizing donor informed consent under Institutional Review Board (IRB) approved oversight by Hummingbird IRB (Needham, MA; STEMCELL Technologies) or Advarra IRB (Columbia, MD; HemaCare Corporation). T cells were thawed and activated the same day. 24 h after activation, lentivirus was mixed with T cells at a final dilution of MOI 6 in 96 well round bottom plates. Per well of the 96 well plate, 1 × 10^6^ T cells were mixed with media containing lentivirus in 200 μl and then spinfected at 1000 g for 60 min. Cells of the same condition were pooled and seeded at 1 × 10^6^ cells/ml in non-TC treated plates. 2 days post transduction, cells transduced with CD47-CAR or CAR-CD47 were collected and pelleted in preparation for nucleofection of Cas9 ribonucleoproteins (RNPs) to generate *TRAC*, *B2M*, and *CIITA* triple knockouts. RNPs for each target locus were complexed separately at a 1.4:1 sgRNA (IDT):SpyFiCas9 (Aldevron) ratio, followed by mixture of the three RNPs at 83.3 pmol TRAC RNP, 125 pmol B2M RNP, and 166.7 pmol of CIITA RNP per million cells. Some allo CAR groups were transduced with CD19-targeting CAR-EGFRt (Figs. 3, 4, 5, and Supplementary Fig. 1), CD19-targeting CAR only (Figs. 1b–d, 2), or CD19-targeting CAR-CD47 (Figs. 6 and 7). The RNP mixture was diluted in P3 solution (Lonza, Basel, Switzerland), mixed with the cell pellet, and nucleofected using the DN-130 program (Lonza). Nucleofected cells were seeded at 1 × 10^6^ cells/ml, split every two days, and cryopreserved 8 days post activation.

### Characterization of T cells

Mock T cells, allo CAR T cells and HIP T cells were characterized in flow cytometry for the T cell markers CD3, CD4 and CD8, for CD19 CAR and CD47, as well as for HLA class I and HLA class II. The following antibodies were used for T cell marker: BV605 conjugated anti-CD3 antibody (clone UCHT1, cat.no. 300460, 1:20 dilution, Biolegend) or IgG1 isotype-matched control antibody (clone MOPC-21, cat.no. 400162, 1:20 dilution, Biolegend), BV421 conjugated anti-CD4 antibody (clone OKT4, cat.no. 317434, 1:20 dilution, Biolegend) or IgG2b isotype-matched control antibody (clone MPC-11, cat.no. 400342, 1:20 dilution, Biolegend), PerCP-Cy5.5 conjugated anti-CD8a antibody (clone HIT8a, cat.no. 300924, 1:20 dilution, Biolegend) or IgG1 isotype-matched control antibody (clone MOPC-21, cat.no. 400150, 1:20 dilution, Biolegend). PE labeled anti-CD19-CAR antibody (clone Y45, cat.no. FM3-PY54A2, 1:50 dilution, Arco Biosystems) or IgG1 isotype-matched control antibody (clone MOPC-21, cat.no. 400140, 1:20 dilution, Biolegend) and FITC conjugated anti-CD47 (clone CC2C6, cat.no. 323106, 1:20 dilution, Biolegend) or IgG1 isotype-matched control antibody (clone MOPC-21, cat.no. 400110, 1:20 dilution, BD Biosciences) were used to assess CD19-CAR and CD47, respectively. To assess HLA expression, cells were labeled with APC labeled anti-HLA-A,B,C antibody (clone G46_2.6, cat.no. 555555, 1:20 dilution, BD Biosciences) or IgG1 isotype-matched control antibody (clone MOPC-21, cat.no. 554681, 1:5 dilution, BD Biosciences) and Alexa-flour647-labeled anti-HLA-DR,DP,DQ antibody (clone Tu39, cat.no. 563591, 1:20 dilution, BD Biosciences) or IgG2a isotype-matched control antibody (clone G155-178, cat.no. 565357, 1:60 dilution, BD Biosciences). To assess CD47 expression, cells were labeled with PerCP-Cy5.5 conjugated anti- CD47 (clone B6H12, cat.no. 561261, 1:20 dilution, BD Biosciences) or IgG1 isotype-matched control antibody (clone MOPC-21, cat.no. 550795, 1:20 dilution, BD Biosciences).

### Exhaustion markers

The expression of the exhaustion markers on allo CAR T cells and HIP CAR T cells was assessed before and after 90 h of incubation with Nalm6 cells, as well as on CAR + cells recovered from the spleen of allo CAR T-treated NSG mice or the HIP CAR T-treated NSG mice after 63 days. The following antibodies were used: TIM3 (BV750, clone F38-2E2, cat.no. 345056, 1:20 dilution) or IgG1 isotype-matched control antibody (clone MOPC-21, cat.no. 400106, 1:20 dilution), TIGIT (APC, clone A15153G, cat.no. 372706, 1:20 dilution), LAG3 (APC, clone MOPC-173, cat.no. 369212, 1:20 dilution), CD39 (FITC, clone A1, cat.no. 328206, 1:20 dilution), CTLA-4 (BV605, clone BNI3, cat.no. 369610, 1:20 dilution) or IgG2a isotype-matched control antibody (clone MOPC-173, cat.no. 400222, 1:20 dilution). PD-1 (FITC, clone A17188A, cat.no. 379206, 1:20 dilution) or IgG2b isotype-matched control antibody (clone MPC-11, cat.no. 400310, 1:20 dilution). All antibodies were from Biolegend. Briefly, cells were incubated with antibodies for 45 min at 4 C in the dark, washed one time with PBS + 2% FCS hi (both Thermo Fisher) and analyzed by flow cytometry (Attune, Thermo Fisher).

The IFN-γ and IL-2 release of HIP CAR T cells and allo CAR T cells is shown after 24 h and 90 h of incubation with Nalm6 in cell culture supernatants using ELISA. ELISA assays of IFN-γ and IL-2 (both Thermo Fisher) were performed according to manufactures protocol. Briefly, standards and samples were added to pre-coated 96-well ELISA plates and incubated for 1 h. After the removal of unbound proteins by washing, IFN-γ or IL-2 antibodies conjugated with horseradish peroxidase were added. These enzyme-labeled antibodies form complexes with the previously bound IFN-γ or IL-2. The enzyme bound to the immunosorbent is assayed by the addition of the chromogenic substrate tetramethylbenzidine. Samples were analyzed in a microplate reader (Thermo Fisher).

### CD47 flow cytometry

CD47 expression (FITC conjugated anti-CD47, clone CC2C6, cat.no. 323106, 1:20 dilution, Biolegend or IgG1 isotype-matched control antibody, clone MOPC-21, cat.no. 400110, 1:20 dilution, BD Biosciences) on primary human fibroblasts, mesenchymal stromal cells, endothelial cells, T cells, and allo CAR T cells. Briefly, cells were incubated with anti-CD47 or IgG1 for 45 min at 4 C in the dark, washed one time with PBS + 2%FCS hi (both Thermo Fisher) and analyzed was assessed by flow cytometry (Attune, Thermo Fisher). Results were shown as fold change to Isotype or as dot plots in FlowJo.

### T cell culture

Human T cells were cultured in Optimizer with T Cell Expansion Supplement and CTS Immune Cell Serum Replacement at 2.5% (Gibco, Carlsbad, CA), 1% Glutamax and 100IU/ml IL-2 (Peprotech, Rocky Hill, NJ) as suspension cells. Media was changed every other day. T cells were sorted for some experiments for CAR positive or negative populations (antibody FMC63, clone Y45, AcroBio Systems, cat.no. FM3-PY54A2, 1:50 dilution, Newark, DE with IgG1, PE, clone MOPC-21, cat.no. 400140, 1:20 dilution, Biolegend isotype control), HLA positive or negative populations (anti-HLA-A,B,C, clone G46_2.6, cat.no. 555555, 1:20 dilution with IgG1, APC, clone MOPC-21, cat.no. 554681, 1:5 dilution isotype control; anti-HLA-DR, DP, DQ, clone Tu39, cat.no. 563591, 1:20 dilution with IgG2a, AF647, clone G155-178, cat.no. 565357, 1:60 dilution isotype control; all BD Biosciences, San Jose, CA) and CD47 positive and negative populations (anti-CD47, clone CC2C6, cat.no. 323106, 1:20 dilution, Biolegend, San Diego, CA with IgG1, FITC, clone MOPC-21, cat.no. 555748, 1:20 dilution, BD Biosciences).

### Cell culture of tumor cells and transduction to express firefly luciferase

Nalm6 fluc+ cells (cat.no. CSC-RR0361, Creative Biogene, Shirly, NY) and Daudi cells (cat.no. CCL-213, ATCC, Manassas, VA) were cultured in non-TC-treated, non-coated T75 flasks in RPMI 1640 plus 10% FCS hi and 1% Pen/Strep as floating cells. Media was changed every other day and split every 3 days. Daudi cells were transduced to express Fluc. One hundred thousand Daudi cells were spinfected using luciferase virus (Gentarget, San Diego, CA) at a MOI of 100 in the presence of 10 µg/ml protamine sulfate at 1000 g for 30 min. Cell cultures were harvested and clarified 2 days post transfection. Luciferase expression was confirmed by adding D-luciferin (Biosynth, Staad, Switzerland). Signals were quantified with Ami HT (Spectral Instruments Imaging, Tuscon, AZ) in maximum photons s^−1^ cm^−2^ sr^–1^.

### Mouse models

Male and female NSG mice (strain# 005557) and female humanized NSG-SGM3 mice (strain# 013062) were purchased from the Jackson Laboratories and used as recipients for different assays. Humanized mice were not thymectomized, received human CD34^+^ cells at 12 weeks of age, and were included into study groups 6–8 weeks after humanization. Animals were randomly assigned to experimental groups irrespective of their sex. The number of animals per experimental group is presented in each figure. Animal experiments were approved by the Explora BioLabs Institutional Animal Care and Use Committee. Animals received humane care and all experiments followed local guidelines. Mice were housed in 12-hour light-dark cycles with humidity between 30–70% at ambient temperature of 20–26 degrees Celsius. The study and control animals were housed in the same room. The animal facility is a specific pathogen-free facility. Health status of mice was monitored as previously described^26^ and mice were individually scored weekly for five clinical parameters (posture, activity, fur, skin and weight loss) to document GVHD^27^. Mice were euthanized when GVHD score was >2. Euthanasia was conducted via exsanguination under isoflurane anesthesia followed by cervical dislocation.

### Bioluminescence imaging

For bioluminescence imaging, D-luciferin firefly potassium salt (375 mg/kg, Biosynth) dissolved in sterile PBS (pH 7.4, Gibco, Invitrogen) was injected intraperitoneally (250 μl per mouse) into anesthetized mice. Animals were imaged using the Ami HT (Spectral Instruments Imaging) ROI bioluminescence was quantified in units of maximum photons per second per centimeter square per steradian (p s^−1^ cm^−2^ sr^−1^). The maximum signal from an ROI was measured using Aura 3.2 software (Spectral Instruments Imaging).

### In vivo BLI experiments

NSG mice or humanized mice were injected with 1 × 10^6^ Nalm6 fluc+ or Daudi fluc+ cells intravenously on day 0. Mice received T cells on day 3 intravenously in an E:T ratio of 1:1, 3:1 or 7:1. Mice were monitored on day 0, day 3 and every 4 days until day 27 or up to 63 days. Some groups received additional Nalm6 Fluc+ cell injection intravenously on day 15 and day 27. Some groups received 5 × 10^6^ NK cells on day 3 and 5 mg of anti-CD47 IgG1 or IgG4 fusion proteins (customized, Creative Biolabs) at a concentration of 5 mg on different days. Some mice served as control animals and did not receive any T cells. The number of animals and different study groups with IgG1 and IgG4 treatment or NK-cell injection is presented in each figure. Splenocytes and bone marrow cells were isolated from all animals for analysis in flow cytometry.

### Flow cytometry of in vivo samples

For the detection of tumor cells and injected CAR T cells, recipient splenocytes and bone marrow cells were stained with Zombie live/dead stain (cat.no. 77184, Biolegend), anti-CD19 (clone HIB19, cat.no. 302212, 1:20 dilution, Biolegend with IgG1, APC, clone MOPC-21, cat.no. 400120, 1:20 dilution, Biolegend) and FMC63 (clone Y45, cat.no. FM3-PY54A2, 1:50 dilution, AcroBio Systems with IgG1, PE, clone MOPC-21, cat.no. 400140, 1:20 dilution, Biolegend). Cells were measured by flow cytometry (BD Aria and FACSDiva version 9.0 or CytExpert version 2.4 software) and gated for live cells, followed by CD19 + cells or CAR + cells. Results were expressed as percentage of positive population compared to control samples in FlowJo (BD).

### Mouse in vivo innate cytotoxicity assay

HIP CAR T cells were sorted for CD47 negative population (dKO CAR-T) and CD47 positive population (HIP CAR-T) using an anti-CD47 antibody (clone CC2C6, cat.no. 323106, 1:20 dilution, Biolegend with IgG1, FITC, clone MOPC-21, cat.no. 555748, 1:20 dilution, BD Biosciences). 5 × 10^6^ human mock transduced T cells and 5 × 10^6^ dKO CAR-T or HIP CAR-T were mixed and injected intraperitoneally into NSG mice. Some mice received 5 mg of anti-CD47 IgG1 or IgG4 fusion proteins (customized, Creative Biolabs) in addition to the HIP CAR-T group. Mock transduced T cells were labeled with DiO and dKO CAR-T or HIP CAR-T were labeled with DiD according to the manufacturer’s protocol (Vybrant Multicolor cell labeling kit; Invitrogen, Carlsbad, CA). After 48 h, cells were collected from the abdomen and analyzed for the percentage of DiO+ and DiD+ cells and compared between the Mock T cells and the CAR-T group by flow cytometry (Attune, Thermo Fisher). All animals were pretreated 18 h with poly I:C (100 μg in sterile PBS i.p.; Sigma-Aldrich, St.Louis, MO) before cell injection.

### Human primary NK-cell and macrophage culture

Human primary peripheral blood NK cells were purchased from Stemcell Technologies (70036) and were stimulated with 1 μg/ml human IL-2 (PeproTech) in RPMI 1640 plus 10% FCS hi and 1% Pen/Strep before performing the killing assays. Cell culture was performed in T25 non-TC-treated, non-coated flasks with a media change every other day. Human primary peripheral macrophages (70042, Stemcell Technologies) were plated in 24-well plates in RPMI 1640 plus 10% FCS hi and 1% Pen/Strep. Attached cells were harvested the next day for subsequent killing assays.

### Characterization of humanized mice in flow cytometry

For the analysis of human cell engraftment in humanized mice, recipient splenocytes and bone marrow cells were stained with Zombie live/dead stain (cat.no. 77184, Biolegend), anti-human CD45 (clone HI30, cat.no. 304008, 1:20 dilution, Biolegend with IgG1, PE, clone MOPC-21, cat.no. 400140 1:20 dilution, Biolegend), anti-human CD3 (clone UCHT1, cat.no. 300415, 1:20 dilution, Biolegend with IgG1, AF488, clone MOPC-21, cat.no. 400129 1:20 dilution, Biolegend), anti-human CD19 (clone HIB19, cat.no. 302228, 1:20 dilution, Biolegend with IgG1, PerCP, clone MOPC-21, cat.no. 400148 1:20 dilution, Biolegend) and anti-human CD33 (clone P67.6, cat.no. 366612, 1:20 dilution, Biolegend with IgG1, BV605, clone MOPC-21, cat.no. 400162 1:20 dilution, Biolegend). Cells were measured by flow cytometry (BD Aria and FACSDiva version 9.0 or CytExpert version 2.4 software) and gated for live cells, CD45 + cells and then followed by CD3 + cells, CD19 + or CD33 + cells. Results were expressed as percentage of positive population compared to control samples in FlowJo (BD).

### Innate killing in vitro by XCelligence

NK cell and macrophage killing assays were performed on the XCelligence MP platform (ACEA BioSciences, San Diego, CA.). Specialized 96-well E-plates (ACEA BioSciences) were coated and 4 × 10^4^ target T cells were plated in 100 μl T cell media. After the Cell Index reached 0.7, effector cells were added at an effector cell to target cell (E:T) ratio of 1:1. NK cells were stimulated with 1 μg /ml human IL-2 (Peprotech). In some wells, an anti-CD47 fusion protein (IgG1 and IgG4, Creative Biolabs) or SIRPa-polyHis protein (Arco Biosystems) were added at a concentration of 100 μg/ml. As killing control, cells were treated with 2% TritonX100 or media only was added (Supplementary Fig. 15). Data were standardized and analyzed with the RTCA software (ACEA).

### T cell killing In vitro by XCelligence

T cell killing assays were performed on the XCelligence MP platform (ACEA BioSciences, San Diego, CA.). Specialized 96-well E-plates (ACEA BioSciences) were coated with tumor coating solution (Agilent) and 4 × 10^4^ target Nalm6 cells or Daudi cells were plated in 100 μl cell-specific media. After the Cell Index reached 0.7, effector T cells were added at different effector cell to target cell (E:T) ratio of 0.125:1 to 7:1. For serial killing XCelligence assays, effector T cells were pooled after 24 h, counted and plated back on a different plate with target cells 4 times in total. As killing control, cells were treated with 2% TritonX100 or media only was added. Data were standardized and analyzed with the RTCA software (ACEA).

### Elispot

For uni-directional Elispot assays, recipient splenocytes were isolated from humanized mice 6 days after intravenous T cell injection and used as responder cells. T cells were sorted for some groups for CAR positive or negative populations (antibody FMC63, clone Y45, AcroBio Systems, cat.no. FM3-PY54A2, 1:50 dilution, Newark, DE with IgG1, PE, clone MOPC-21, cat.no. 400140, 1:20 dilution, Biolegend isotype control) and HLA negative populations (anti-HLA-A,B,C, clone G46_2.6, cat.no. 555555, 1:20 dilution with IgG1, APC, clone MOPC-21, cat.no. 554681, 1:5 dilution isotype control; anti-HLA-DR, DP, DQ, clone Tu39, cat.no. 563591, 1:20 dilution with IgG2a, AF647, clone G155-178, cat.no. 565357, 1:60 dilution isotype control; all BD Biosciences, San Jose, CA). Donor cells were mitomycin-treated (50 μg/ml for 30 min) and used as stimulator cells. 1 × 10^5^ stimulator cells were incubated with 1 × 10^6^ recipient responder splenocytes for 48 h and IFN-γ spot frequencies were enumerated using an Elispot plate reader.

### Splenocyte killing in XCelligence

Splenocyte killing assays were performed on the XCelligence MP platform. Specialized 96-well E-plates (ACEA BioSciences) were coated and 4 × 10^4^ target T cells were plated in 100 μl T cell media. After the Cell Index reached 0.7, effector cells were added at an effector cell to target cell (E:T) ratio of 1:1. As killing control, cells were treated with 2% TritonX100 or media only was added. Data were standardized and analyzed with the RTCA software (ACEA).

### Statistics

All data are expressed as mean ± SD or mean ± SEM. Intergroup differences between two groups were assessed by unpaired two-tailed Student’s *t* test. Statistical analyses of three or more groups were performed using a one-way ANOVA followed by the Bonferroni post hoc test. GraphPad Prism 9 was used for all analyses.

### Reporting summary

Further information on research design is available in the Nature Portfolio Reporting Summary linked to this article.

## Supplementary information

Supplementary Information

Reporting Summary

## Data Availability

The authors declare that all data supporting the findings of this study are available within the paper and its Supplementary Information files. Correspondence and requests for materials should be addressed to S.S. (sonja.schrepfer@sana.com). The raw numbers for charts and graphs are available in the Source Data file whenever possible. Source data are provided with this paper.

## Source data

Source Data File
