# Supplementary material for: Hypoimmune anti-CD19 chimeric antigen receptor T cells provide lasting tumor control in fully immunocompetent allogeneic humanized mice
Source: Nat Commun. 2023 Apr 10;14:2020. doi: 10.1038/s41467-023-37785-2 (PMC10086027; doi:10.1038/s41467-023-37785-2)
Supplement: Supplementary file 1 — Supplementary Information [file 41467_2023_37785_MOESM1_ESM.pdf]

Hypoimmune anti-CD19 chimeric antigen receptor T cells provide lasting tumor control in fully immunocompetent allogeneic humanized mice

Xiaomeng Hu[1], Karl Manner[1], Rowena DeJesus[1], Kathy White[1], Corie Gattis[1], Priscilla Ngo[1], Christopher Bandoro[1], Eleonore Tham[1], Elaine Y. Chu[1], Chi Young[1], Frank Wells[1], Ronald Basco[1], Annabelle Friera[1], Divy Kangeyan[1], Pascal Beauchesne[1], William E. Dowdle[1], Tobias Deuse[2], Terry J Fry[1], Aaron E. Foster[1], Sonja Schrepfer[1#]

[1]Sana Biotechnology Inc., 1 Tower Place, South San Francisco, CA, USA.
[2]Present address Department of Surgery, Division of Cardiothoracic Surgery, Transplant and Stem Cell Immunobiology (TSI)-Lab, University of California San Francisco, San Francisco, California, USA.

#Correspondence should be addressed to S.S. (Sonja.Schrepfer@sana.com)

Supplementary Figures

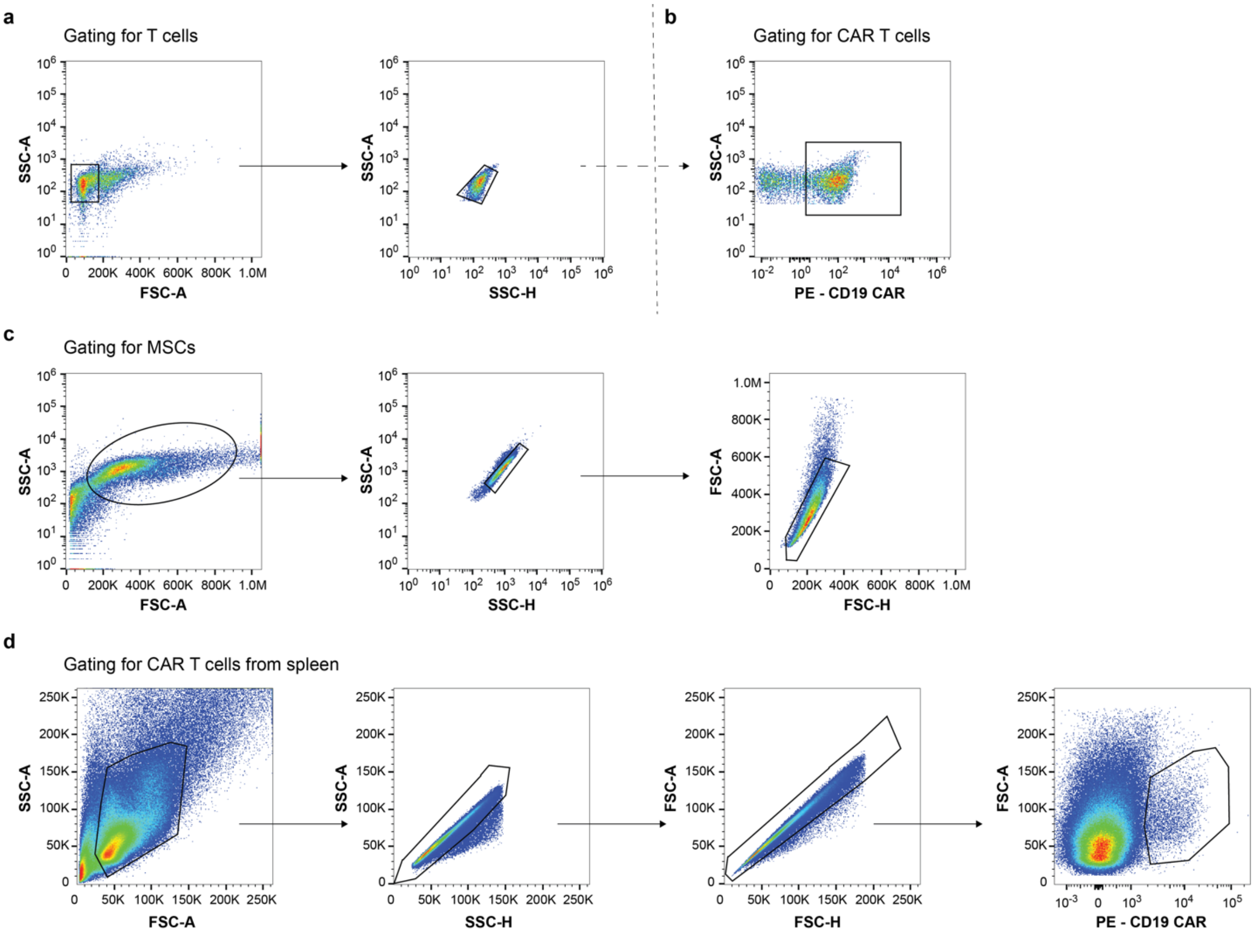

Supplementary Figure 1: *Gating strategy for flow cytometry*. **a**, One example showing the gating strategy for human T cells (Fig. 1a). **b**, When gating CAR T cells, the gating shown in (a) is used and one additional gating step for the CD19 CAR is included (Fig. 1b-c, Suppl. Fig. 5a-d, Suppl. Fig. 10b, Suppl. Fig. 11b). **c**, One example showing the gating strategy for human MSCs, which is similar to those of other benign cell types (Suppl. Fig. 10a, Suppl. Fig. 11a). **d**, One example showing the gating strategy for CAR T cells isolated from spleen of mice (Suppl. Fig. 7).

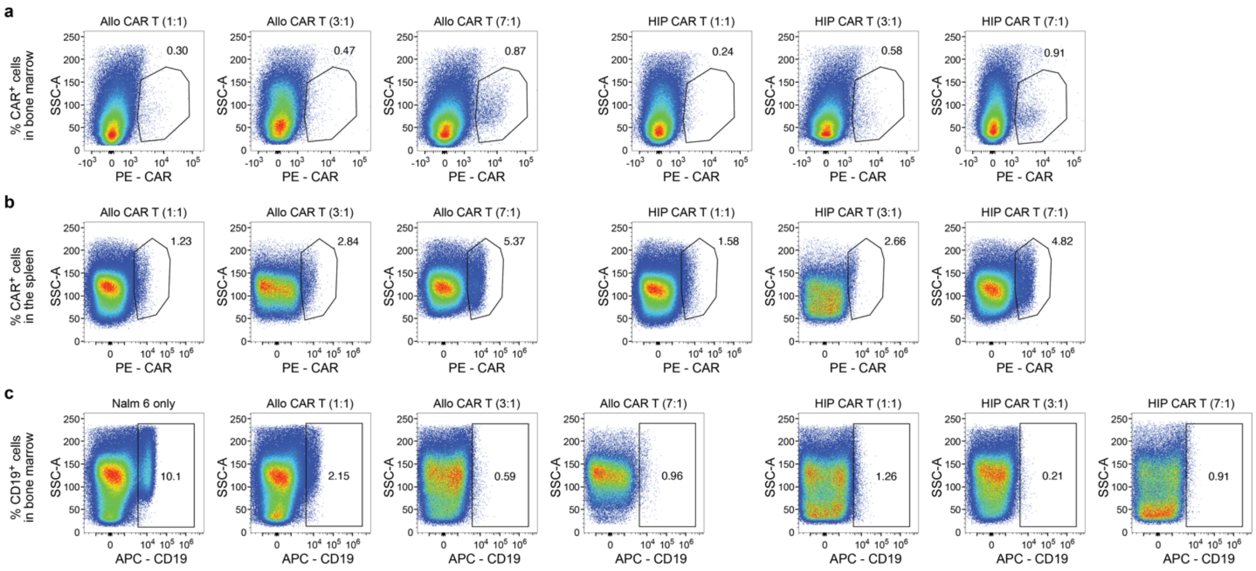

Supplementary Figure 2: *Additional flow cytometry dot plots for Figure 2*. One representative flow cytometry dot plot per groups is shown for Figure 2e (a), Figure 2f (b), and Figure 2g (c). The percentages of cells positive for any specific marker are presented.

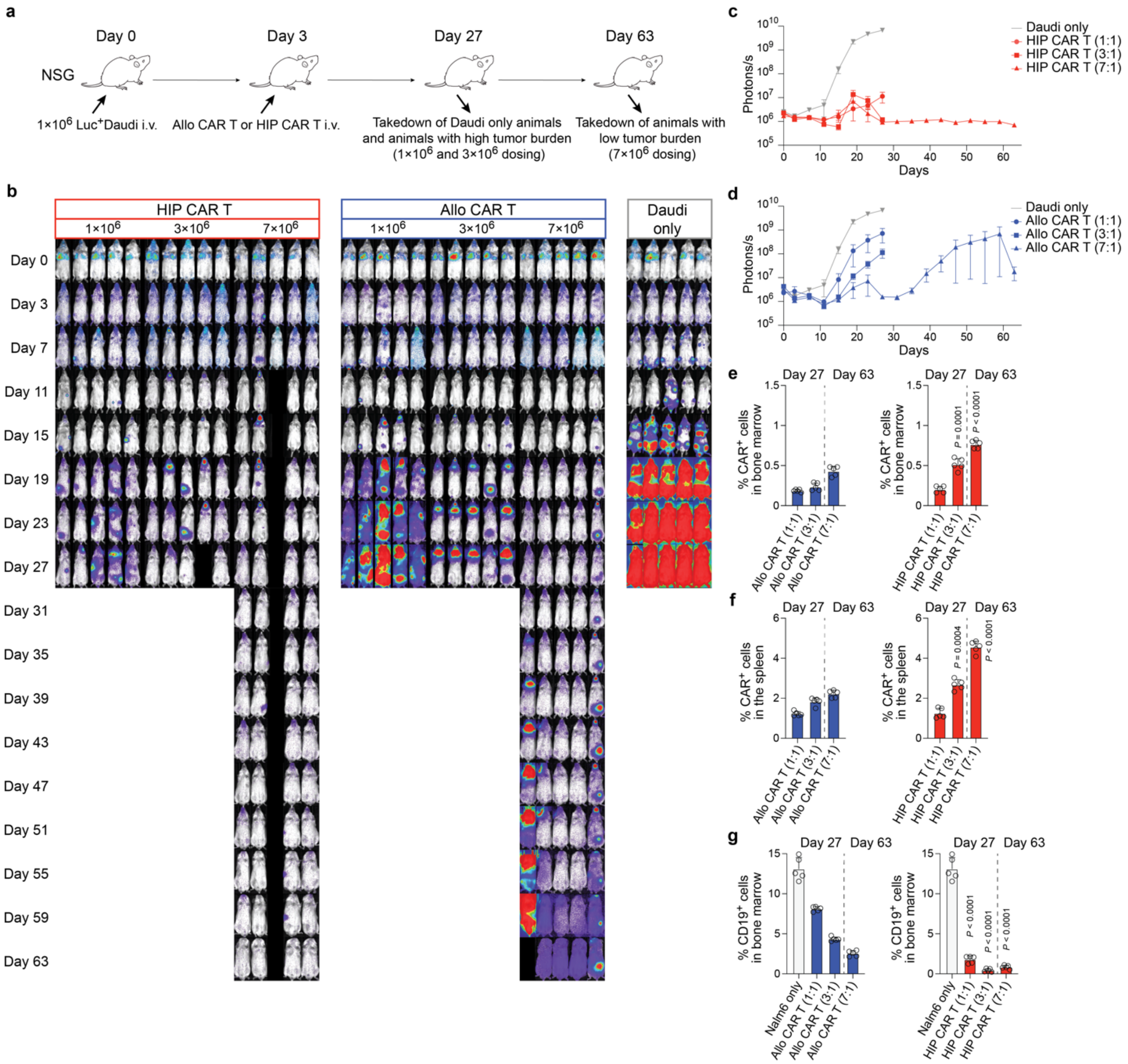

Supplementary Figure 3: *Daudi tumor killing in NSG mice*. **a**, Immunodeficient NSG mice were injected with 1×10^6 Luc+ Daudi cells via the tail vein and were monitored using BLI. Some mice then received allo CAR T cells or HIP CAR T cells intravenously on day 3. Spleen and bone marrow were taken after 27 or 63 days. **b**, BLI images show the tumor burden for all mice in this study. **c-d**, Graphs show BLI signals for animals in the HIP CAR T (c) and allo CAR T cell groups (d, mean ± SEM, n=5 animals per group). **e-f**, The percentage of CAR+ cells in the bone marrow (e) and spleen (f) were assessed on the day of organ recovery (mean ± SD, n=5 animals per group). Significant differences between the two CAR T cell groups at the same dose level are shown. **g**, The percentage of CD19+ cells in the bone marrow was assessed on the day of recovery (mean ± SD, n=5 animals per group). For (e-g): Unpaired, two-tailed Student's t test was used to compare CAR T cell groups at the same dose level and significant differences are shown.

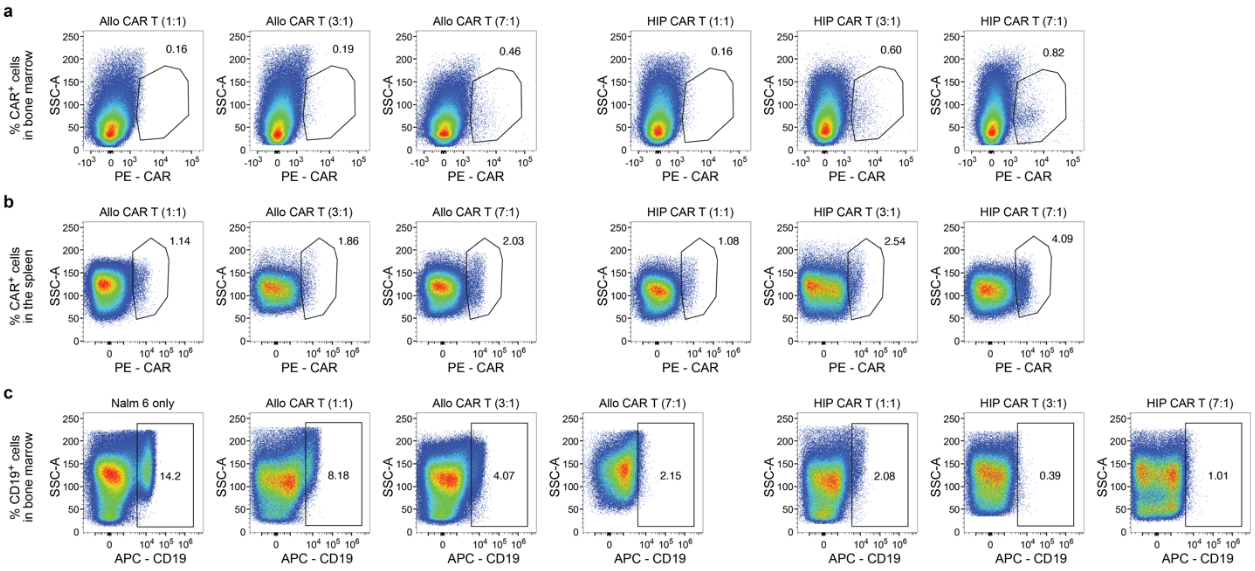

Supplementary Figure 4: *Additional flow cytometry dot plots for Supplementary Figure 3*. One representative flow cytometry dot plot per groups is shown for Supplementary Figure 2e (a), Supplementary Figure 2f (b), and Supplementary Figure 2g (c). The percentages of cells positive for any specific marker are presented.

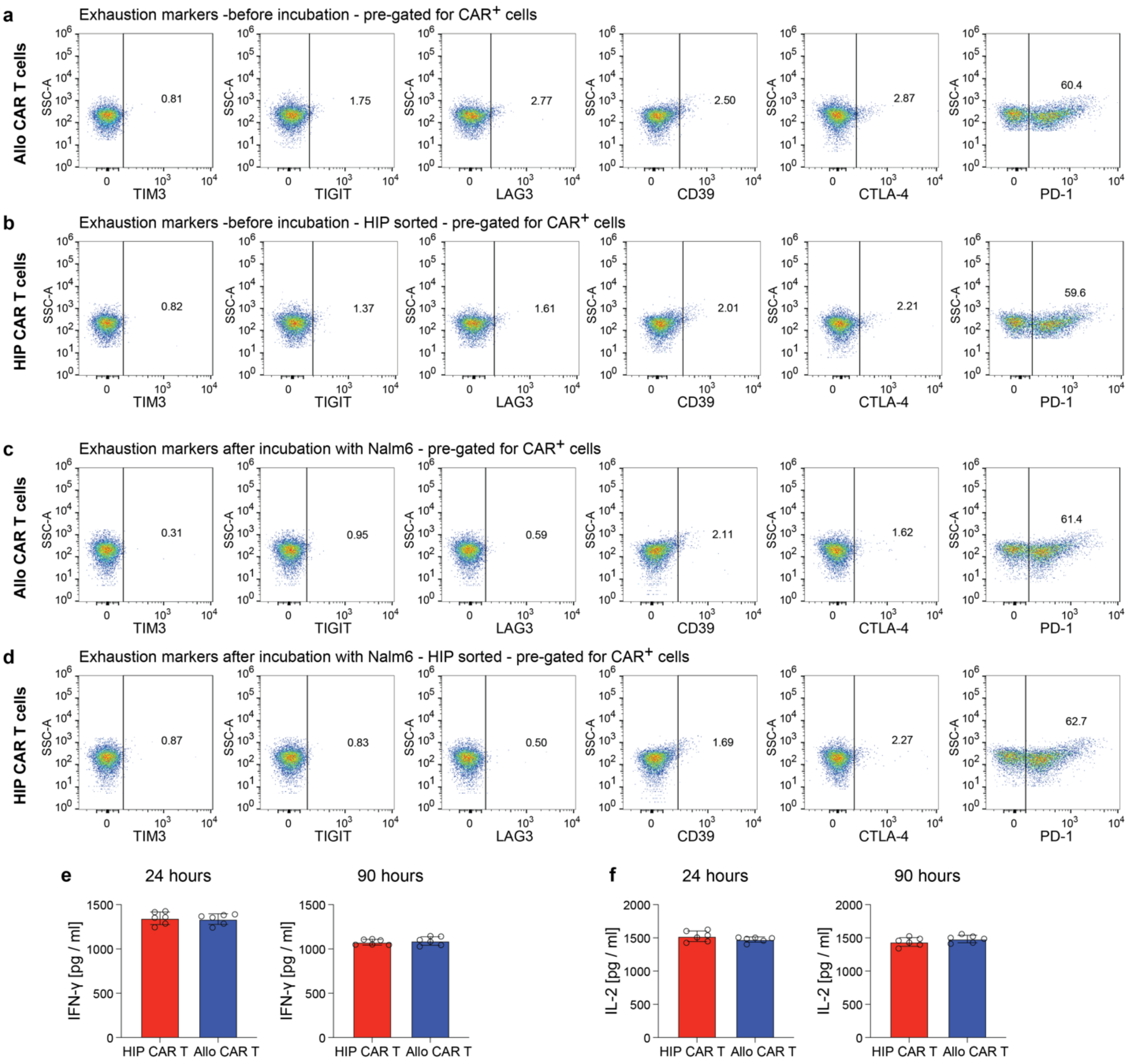

Supplementary Figure 5: *Exhaustion of CAR T cells in vitro*. **a-d**, The expression of the exhaustion markers TIM3, TIGIT, LAG3, CD39, CTLA-4, and PD-1 on allo CAR T cells (a-b) and HIP CAR T cells (c-d) was assessed before (a, c) and after 90 hours of incubation with Nalm6 cells (b, d). Shown are representative flow cytometry dot plots of three independent experiments. The percentage of positive cells is shown. **e-f**, The IFN-γ (e) and IL-2 release capacity (f) of HIP CAR T cells and allo CAR T cells is shown after 24 hours and 90 hours of incubation with Nalm6 (mean ± SD, n=6 per group, unpaired, two-tailed Student's t test was used to compare HIP and allo CAR T cell groups and no significant differences were found).

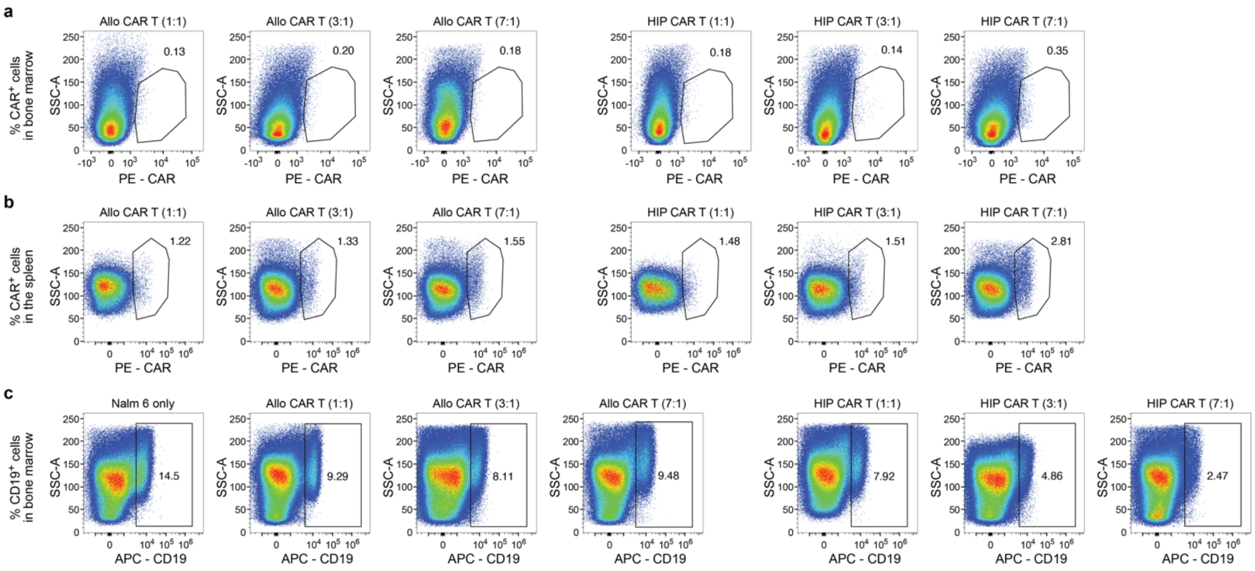

Supplementary Figure 6: *Additional flow cytometry dot plots for Figure 4*. One representative flow cytometry dot plot per groups is shown for Figure 4e (a), Figure 4f (b), and Figure 4g (c). The percentages of cells positive for any specific marker are presented.

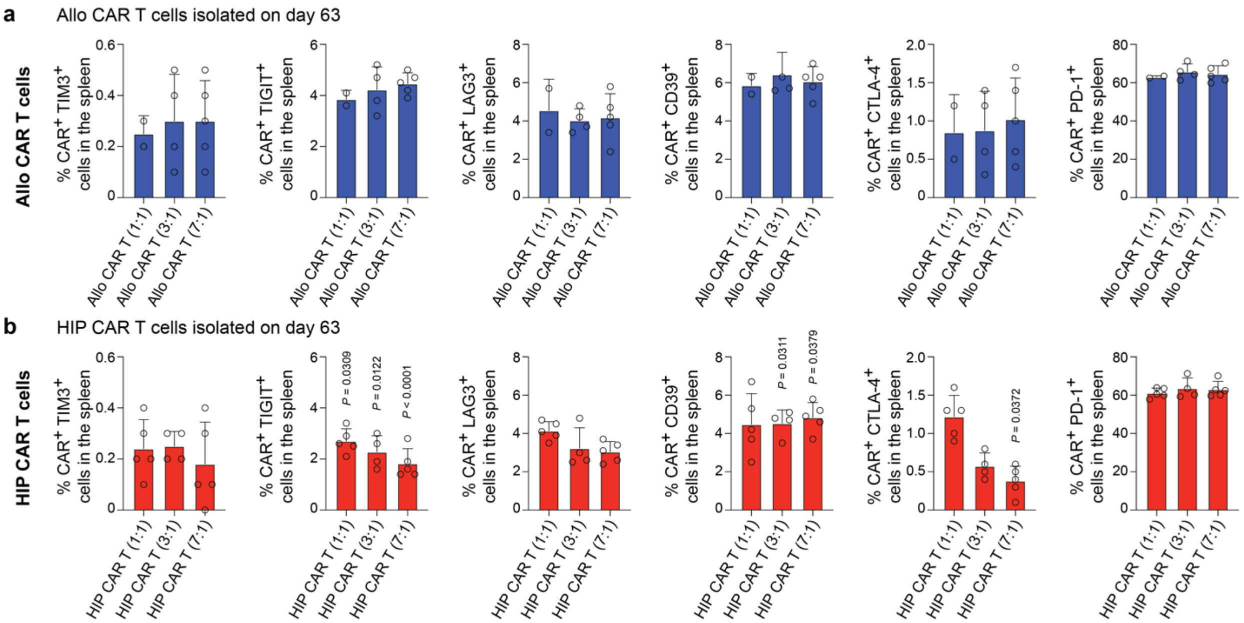

Supplementary Figure 7: *Exhaustion of CAR T cells in vivo*. The CAR^{+} cells recovered from the spleen of allo CAR T-treated NSG mice (a) or the HIP CAR T-treated NSG mice (b) after 63 days were assessed for their expression of TIM3, TIGIT, LAG3, CD39, CTLA-4, and PD-1 (mean ± SD, n=2 in the allo CAR T (1:1) group, n=4 in the allo CAR T (3:1) group, n=5 in the allo CAR T (7:1) group, n=5 in the HIP CAR T (1:1) group, n=4 in the HIP CAR T (3:1) group, n=5 in the HIP CAR T (7:1) group, unpaired, two-tailed Student's t test was used to compare CAR T cell groups at the same dose level and significant differences are shown).

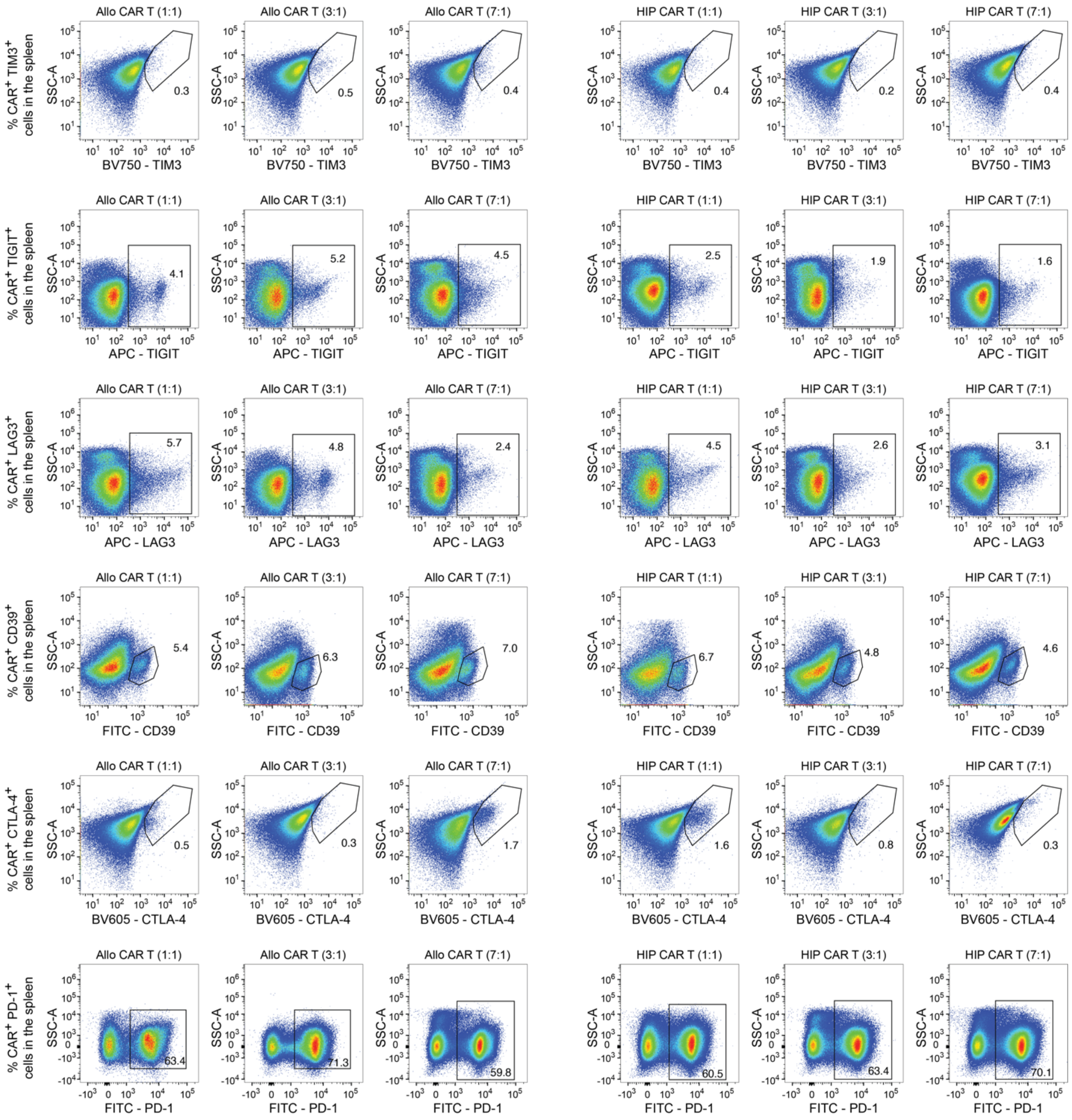

Supplementary Figure 8: *Additional flow cytometry dot plots for Supplementary Figure 7*. One representative flow cytometry dot plot per groups is shown. The percentages of cells positive for any specific marker are presented.

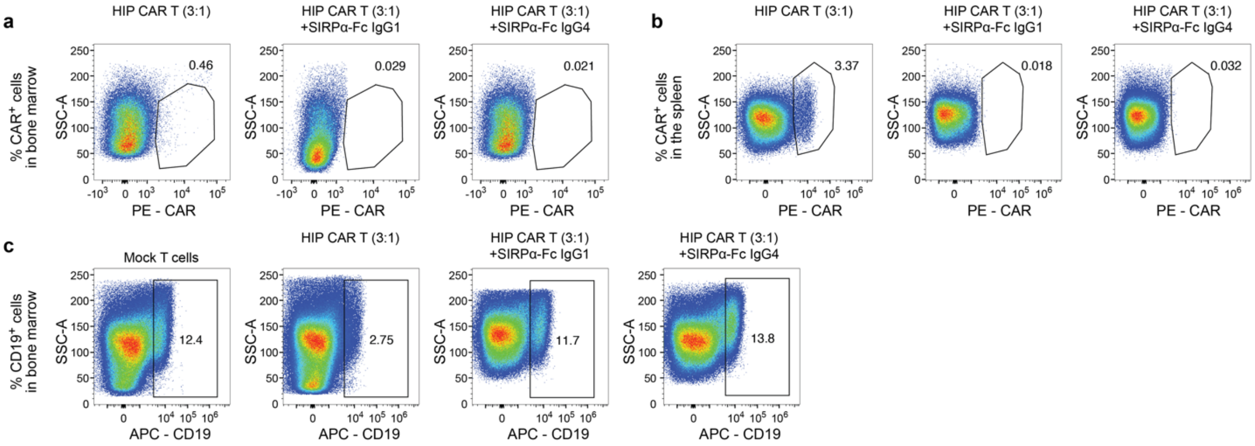

Supplementary Figure 9: *Additional flow cytometry dot plots for Figure 5*. One representative flow cytometry dot plot per groups is shown for Figure 5f (a), Figure 5g (b), and Figure 5h (c). The percentages of cells positive for any specific marker are presented.

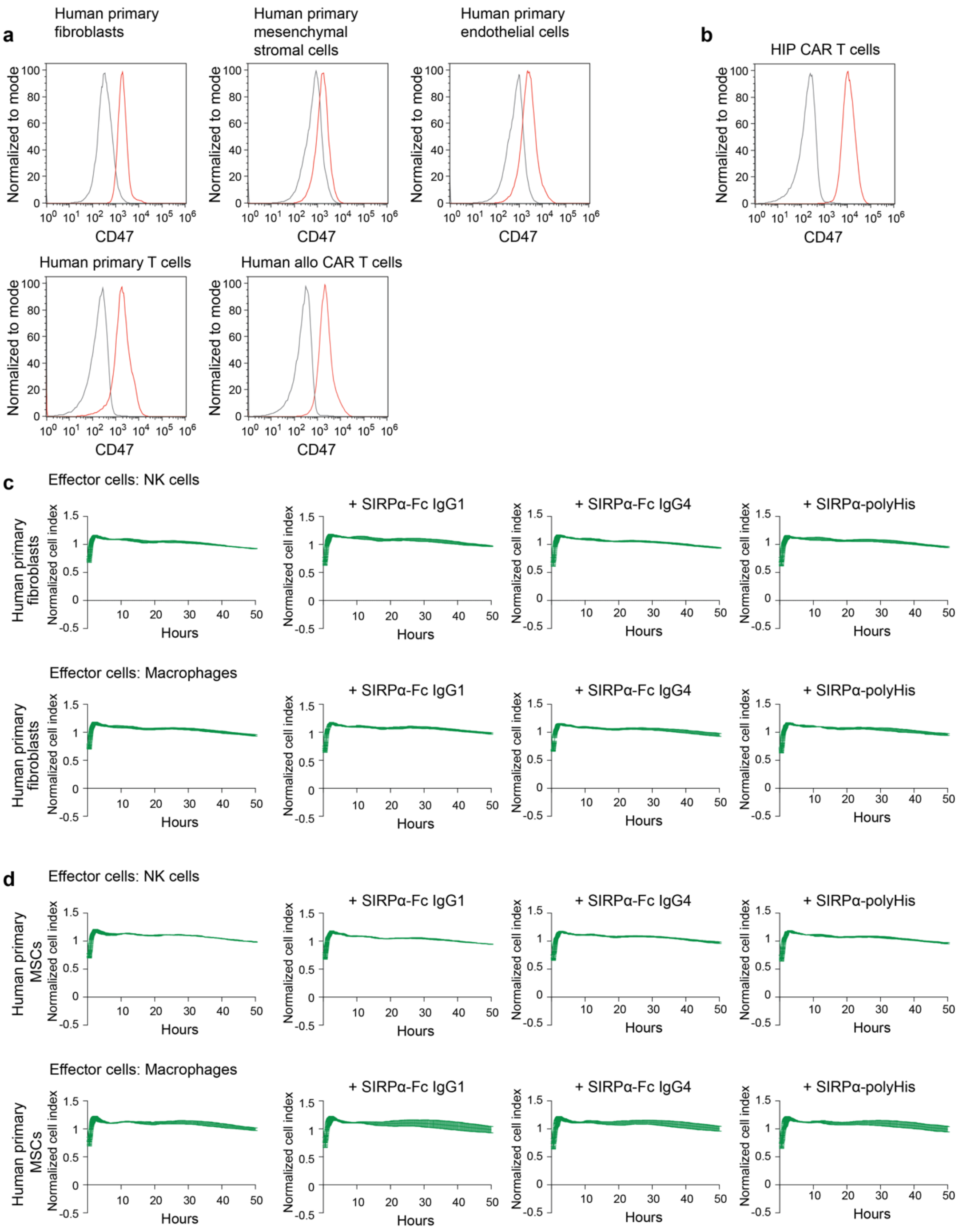

Supplementary Figure 10: *Off-target cytotoxicity of the CD47-targeting safety strategy*. **a-b**, CD47 expression on primary human fibroblasts, mesenchymal stromal cells, endothelial cells, T cells, and allo CAR T cells was assessed by flow cytometry (a) and is shown in comparison with that on HIP CAR T cells (b). Shown are representative flow cytometry histograms of two independent experiments. **c-d**, *In vitro* cytotoxicity assays with NK cells and macrophages as effector cells and SIRPα-Fc IgG1, SIRPα-Fc IgG4, or SIRPα-polyHis are shown (mean ± SD, three independent replicates per group and time point). Target cells were primary human fibroblasts (c) or human primary mesenchymal stromal cells (d).

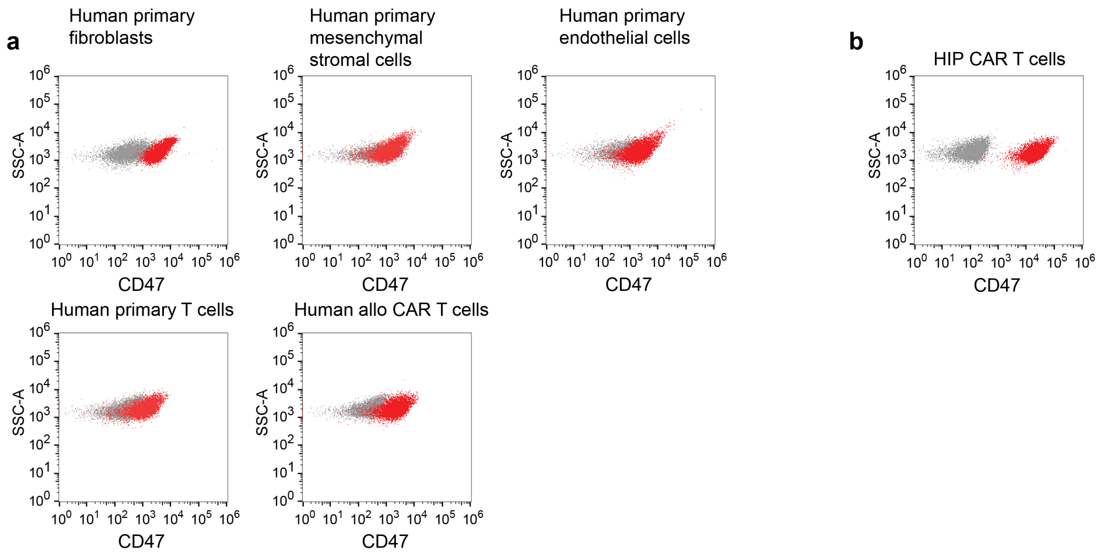

Supplementary Figure 11: *CD47 expression on various cell populations*. **a-b**, CD47 expression on primary human fibroblasts, mesenchymal stromal cells, endothelial cells, T cells, and allo CAR (a) as well as on HIP CAR T cells (b) is shown as dot plots. Representative flow cytometry dot plots of two independent experiments.

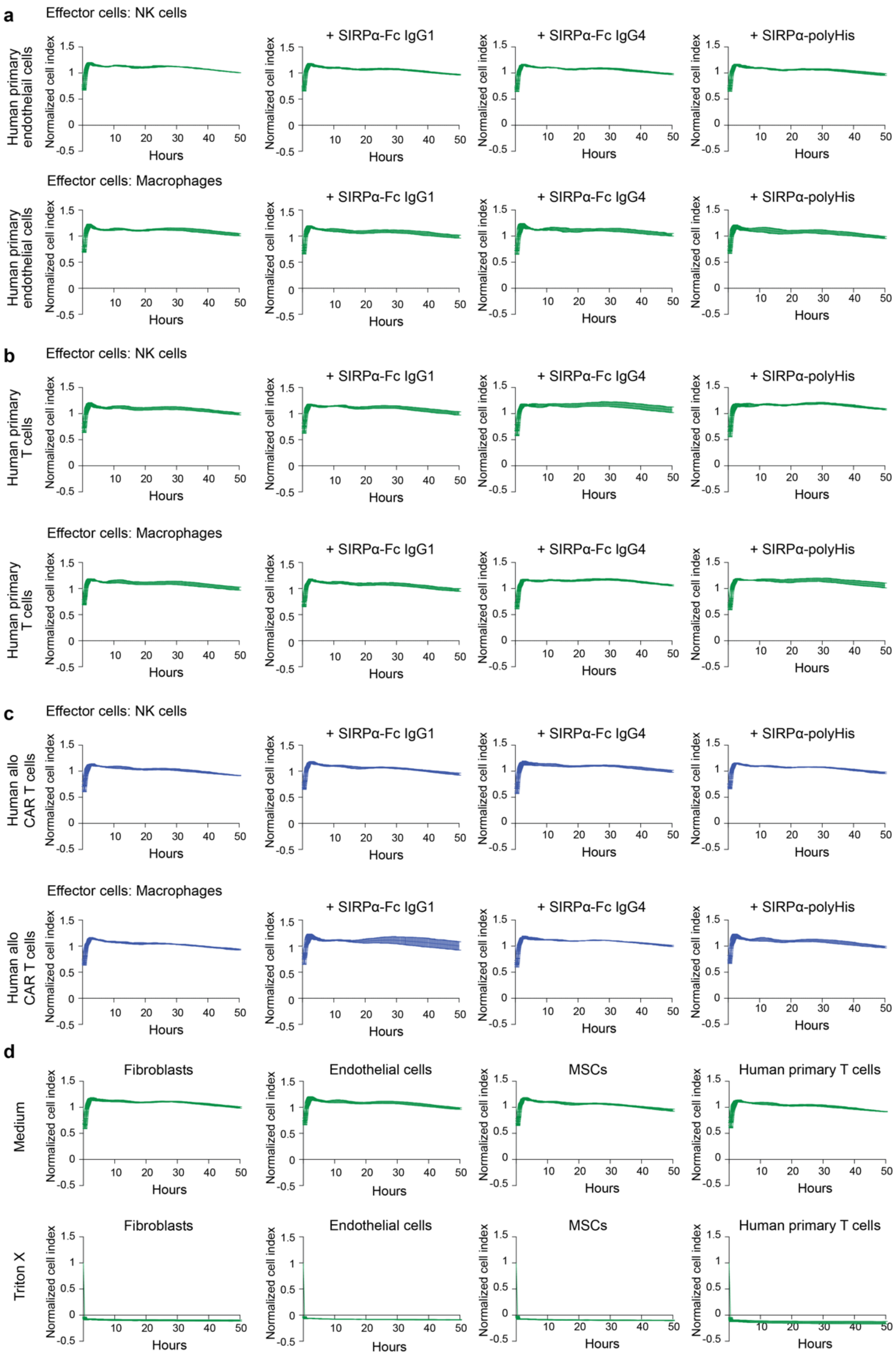

Supplementary Figure 12: *Off-target cytotoxicity of the CD47-targeting safety strategy*. **a-c**, *In vitro* cytotoxicity assays with NK cells and macrophages as effector cells and SIRPα-Fc IgG1,

SIRPα-Fc IgG4, or SIRPα-polyHis are shown (mean ± SD, three independent replicates per group and time point). Target cells were primary human endothelial cells (a), T cells (b), or allo CAR T cells (c). **d**, Fibroblasts, endothelial cells, MSCs, and human primary T cells were grown in medium only or treated with Triton X (mean ± SD, three independent replicates per group and time point). The groups belong to Supplementary Figs. 5 and 7.

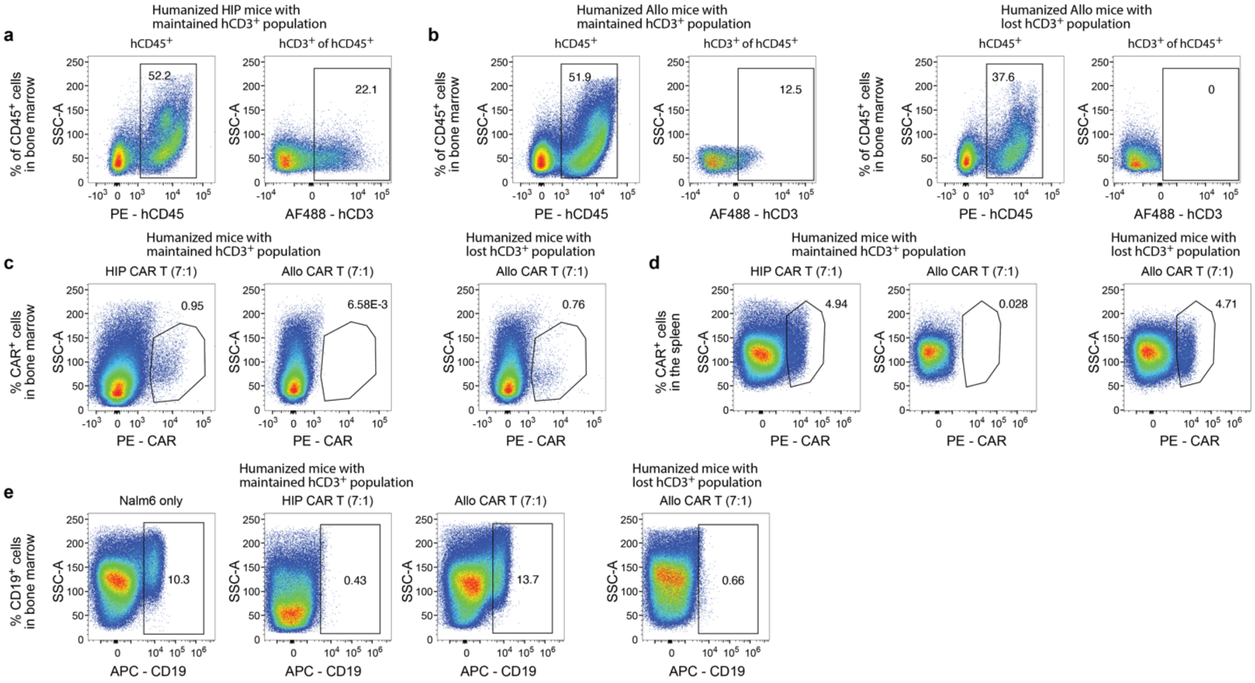

Supplementary Figure 13: *Additional flow cytometry dot plots for Figure 7*. One representative flow cytometry dot plot per groups is shown for Figure 7i (a), Figure 7j (b), and Figure 7f, k (c), Figure 7g, l (d), and Figure 7h, m (e). The percentages of cells positive for any specific marker are presented.

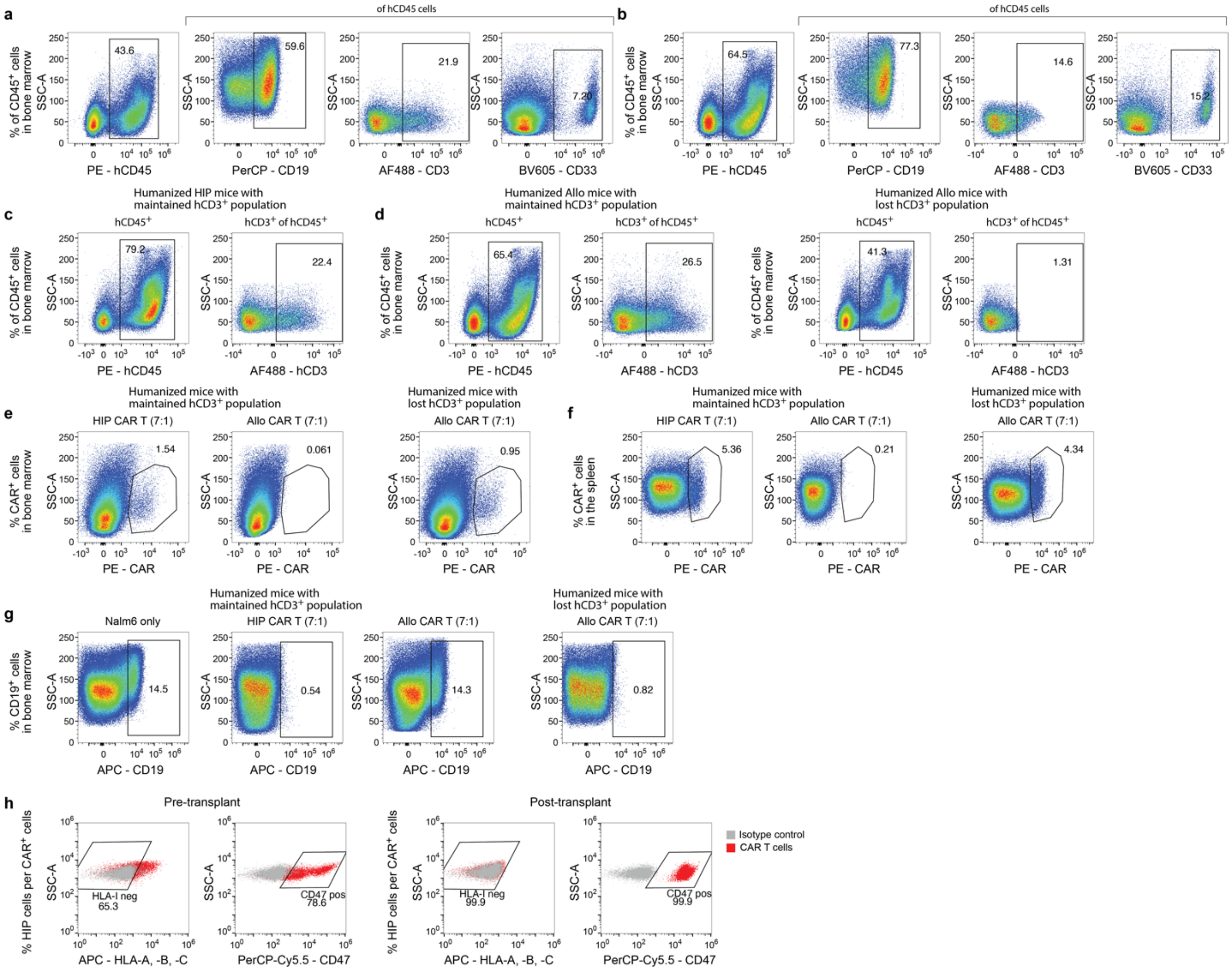

Supplementary Figure 14: *Additional flow cytometry dot plots for Figure 8*. One representative flow cytometry dot plot per groups is shown for Figure 8b (a), Figure 8c (b), and Figure 8h (c), Figure 8i (d), and Figure 8j (e), Figure 8k (f), Figure 8l (g), and Figure 8m (h). The percentages of cells positive for any specific marker are presented.

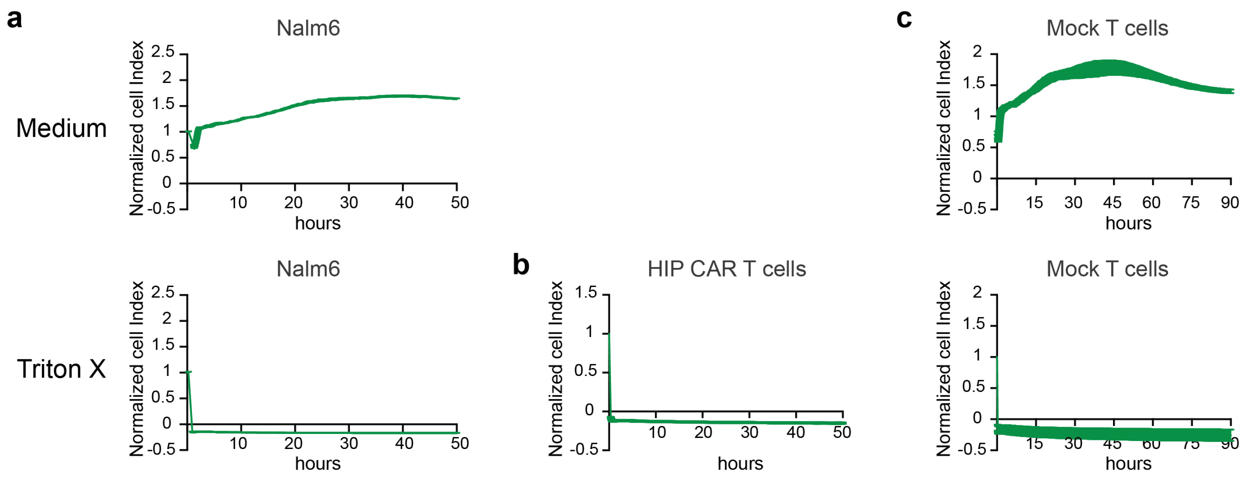

Supplementary Figure 15: *Control groups for XCelligence assays*. **a**, Nalm6 tumor cells were grown in medium only or treated with Triton X (mean ± SD, three independent replicates per group and time point). The groups belong to Fig. 1d-f. **b**, HIP CAR T cells were treated with Triton X (mean ± SD, three independent replicates per time point). The groups belong to Fig. 5a-b. **c**, Mock T cells were grown in medium only or treated with Triton X (mean ± SD, three independent replicates per group and time point). The groups belong to Fig. 6.

Supplementary Tables
Supplementary Table 1: Antibodies used in this study.

antibody	fluorochrome	clone	company	cat.no.	dilution
HLA-A,B,C	APC	G46_2.6	BD Biosciences	555555	1:20
IgG1	APC	MOPC-21	BD Biosciences	554681	1:5
HLA-DR,DP,DQ	AF647	Tu39	BD Biosciences	563591	1:20
IgG2a	AF647	G155-178	BD Biosciences	565357	1:60
CD47	FITC	CC2C6	Biolegend	323106	1:20
IgG1	FITC	MOPC-21	Biolegend	400110	1:20
CD47	PerCP/Cy5.5	B6H12	BD Biosciences	561261	1:20
IgG1	PerCP/Cy5.5	MOPC-21	BD Biosciences	550795	1:20
CD19-CAR	PE	Y45	Arco Biosystems	FM3-PY54A2	1:50
IgG1	PE	MOPC-21	Biolegend	400140	1:20
CD3	BV605	UCHT1	Biolegend	300460	1:20
IgG1	BV605	MOPC-21	Biolegend	400162	1:20
CD4	BV421	OKT4	Biolegend	317434	1:20
IgG2b	BV421	MPC-11	Biolegend	400342	1:20
CD8a	PerCP-Cy5.5	HIT8a	Biolegend	300924	1:20
IgG1	PerCP-Cy5.5	MOPC-21	Biolegend	400150	1:20
CD19	APC	HIB19	Biolegend	302212	1:20
IgG1	APC	MOPC-21	Biolegend	400120	1:20
TIM3	BV750	F38-2E2	Biolegend	345056	1:20
IgG1	BV750	MOPC-21	Biolegend	400106	1:20
TIGIT	APC	A15153G	Biolegend	372706	1:20
IgG2a	APC	MOPC-173	Biolegend	400222	1:20
LAG3	APC	7H2C65	Biolegend	369212	1:20
CD39	FITC	A1	Biolegend	328206	1:20
CTLA-4	BV605	BNI3	Biolegend	369610	1:20
IgG2a	BV605	MOPC-173	Biolegend	400270	1:20
PD-1	FITC	A17188A	Biolegend	379206	1:20
IgG2b	FITC	MPC-11	Biolegend	400310	1:20

Supplementary Table 1 (cont.): Antibodies used in this study.

antibody	fluorochrome	clone	company	cat.no.	dilution
CD45	PE	HI30	Biolegend	304008	1:20
CD3	AF488	UCHT1	Biolegend	300415	1:20
IgG1	AF488	MOPC-21	Biolegend	400129	1:20
CD19	PerCP	HIB19	Biolegend	302228	1:20
IgG1	PerCP	MOPC-21	Biolegend	400148	1:20
CD33	BV605	P67.6	Biolegend	366612	1:20
IgG1	BV605	MOPC-21	Biolegend	400162	1:20
