# Supplementary material for: Hypoimmune anti-CD19 chimeric antigen receptor T cells provide lasting tumor control in fully immunocompetent allogeneic humanized mice
Source: Nat Commun. 2023 Apr 10;14:2020. doi: 10.1038/s41467-023-37785-2 (PMC10086027; doi:10.1038/s41467-023-37785-2)
Supplement: Supplementary file 2 — Reporting Summary [file 41467_2023_37785_MOESM2_ESM.pdf]

nature portfolio

Corresponding author(s): Sonja Schrepfer

Last updated by author(s): Mar 1, 2023

Reporting Summary

Nature Portfolio wishes to improve the reproducibility of the work that we publish. This form provides structure for consistency and transparency in reporting. For further information on Nature Portfolio policies, see our Editorial Policies and the Editorial Policy Checklist.

Statistics

For all statistical analyses, confirm that the following items are present in the figure legend, table legend, main text, or Methods section.

n/a	Confirmed	
☐	☒	The exact sample size (n) for each experimental group/condition, given as a discrete number and unit of measurement
☐	☒	A statement on whether measurements were taken from distinct samples or whether the same sample was measured repeatedly
☐	☒	The statistical test(s) used AND whether they are one- or two-sided<br>*Only common tests should be described solely by name; describe more complex techniques in the Methods section.*
☒	☐	A description of all covariates tested
☒	☐	A description of any assumptions or corrections, such as tests of normality and adjustment for multiple comparisons
☐	☒	A full description of the statistical parameters including central tendency (e.g. means) or other basic estimates (e.g. regression coefficient) AND variation (e.g. standard deviation) or associated estimates of uncertainty (e.g. confidence intervals)
☐	☒	For null hypothesis testing, the test statistic (e.g. F, t, r) with confidence intervals, effect sizes, degrees of freedom and P value noted<br>*Give P values as exact values whenever suitable.*
☒	☐	For Bayesian analysis, information on the choice of priors and Markov chain Monte Carlo settings
☒	☐	For hierarchical and complex designs, identification of the appropriate level for tests and full reporting of outcomes
☒	☐	Estimates of effect sizes (e.g. Cohen's d, Pearson's r), indicating how they were calculated

Our web collection on statistics for biologists contains articles on many of the points above.

Software and code

Policy information about availability of computer code

Data collection	Flow data were collected on BDFACS Diva 9.0, CytExpert Software 2.4 or AttuneNxT software 5.1. Aura 3.2 was used for data collection of bioluminescence imaging. Elispots were enumerated by Immunospot 7.0 software. XCelligence assays were measured with the RTCA 2.1 software.
Data analysis	Data was automatically analyzed in the mentioned software above for RTCA version 2.1. and Immunospot version 7.0.. Statistical analysis and graphing was performed on Prism9. FlowJo10 was used to analyze flow cytometric data. Aura 3.2 was used for quantification of bioluminescence imaging.

For manuscripts utilizing custom algorithms or software that are central to the research but not yet described in published literature, software must be made available to editors and reviewers. We strongly encourage code deposition in a community repository (e.g. GitHub). See the Nature Portfolio guidelines for submitting code & software for further information.

Data

Policy information about availability of data

All manuscripts must include a data availability statement. This statement should provide the following information, where applicable:
- Accession codes, unique identifiers, or web links for publicly available datasets
- A description of any restrictions on data availability
- For clinical datasets or third party data, please ensure that the statement adheres to our policy

The authors declare that all data supporting the findings of this study are available within the paper and its Supplementary Information files.

Human research participants

Policy information about studies involving human research participants and Sex and Gender in Research.

Reporting on sex and gender	no human research participants were involved
Population characteristics	no human research participants were involved
Recruitment	no human research participants were involved
Ethics oversight	no human research participants were involved

Note that full information on the approval of the study protocol must also be provided in the manuscript.

Field-specific reporting

Please select the one below that is the best fit for your research. If you are not sure, read the appropriate sections before making your selection.

☒ Life sciences ☐ Behavioural & social sciences ☐ Ecological, evolutionary & environmental sciences

For a reference copy of the document with all sections, see nature.com/documents/nr-reporting-summary-flat.pdf

Life sciences study design

All studies must disclose on these points even when the disclosure is negative.

Sample size	The sample size for the in vivo studies to achieve statistical significance was not calculated before the studies as the efficiency of hypoimmune CAR T cells in the different models was unknown prior. It was reasoned that 5 mice per group in individual experiments would indicate valid efficacy. Sample sizes in vitro were determined by three or more samples for comparisons between one or multiple groups, followed by the statistical test.
Data exclusions	No pre-established data exclusion method was used.
Replication	The experimental findings can be reliably reproduced. Some key data generated by one co-author were repeated by other co-authors. The figure legends specify how often the experiments were replicated or performed independently.
Randomization	All samples were number coded until the readout was finalized. The numbers were assigned prior to the experiment and determined the group/ treatment/ condition. Animals were number coded and randomly assigned to a group prior to the surgical procedure.
Blinding	Group allocation for cell transplantations were performed by blinded investigators. The investigators doing the readouts were usually blinded, unless the expertise for the readouts overlapped with the data collection.

Reporting for specific materials, systems and methods

We require information from authors about some types of materials, experimental systems and methods used in many studies. Here, indicate whether each material, system or method listed is relevant to your study. If you are not sure if a list item applies to your research, read the appropriate section before selecting a response.

Materials & experimental systems

n/a	Involved in the study	
☐	☒	Antibodies
☐	☒	Eukaryotic cell lines
☒	☐	Palaeontology and archaeology
☐	☒	Animals and other organisms
☒	☐	Clinical data
☒	☐	Dual use research of concern

Methods

n/a	Involved in the study	
☒	☐	ChIP-seq
☐	☒	Flow cytometry
☒	☐	MRI-based neuroimaging

Antibodies

Antibodies used

HLA-A,B,C APC G46_2.6 BD Biosciences 555555 1:20
IgG1 APC MOPC-21 BD Biosciences 554681 1:5
HLA-DR,DP,DQ AF647 Tu39 BD Biosciences 563591 1:20
IgG2a AF647 G155-178 BD Biosciences 565357 1:60
CD47 FITC CC2C6 Biolegend 323106 1:20
IgG1 FITC MOPC-21 BD Biosciences 555748 1:20

CD19-CAR PE Y45 Arco Biosystems FM3-PY54A2 1:50
IgG1 PE MOPC-21 Biolegend 400140 1:20
CD3 BV605 UCHT1 Biolegend 300460 1:20
IgG1 BV605 MOPC-21 Biolegend 400162 1:20
CD4 BV421 OKT4 Biolegend 317434 1:20
IgG2b BV421 MPC-11 Biolegend 400342 1:20
CD8a PerCP-Cy5.5 HIT8a Biolegend 300924 1:20
IgG1 PerCP-Cy5.5 MOPC-21 Biolegend 400150 1:20
CD19 APC HIB19 Biolegend 302212 1:20
IgG1 APC MOPC-21 Biolegend 400120 1:20

TIM3 BV750 F38-2E2 Biolegend 345056 1:20
IgG1 BV750 MOPC-21 Biolegend 400106 1:20
TIGIT APC A15153G Biolegend 372706 1:20
IgG2a APC MOPC-173 Biolegend 400222 1:20
LAG3 APC 7H2C65 Biolegend 369212 1:20
CD39 FITC A1 Biolegend 328206 1:20
CTLA-4 BV605 BNI3 Biolegend 369610 1:20
IgG2a BV605 MOPC-173 Biolegend 400270 1:20
PD-1 FITC A17188A Biolegend 379206 1:20
IgG2b FITC MPC-11 Biolegend 400310 1:20

Validation

Each antibody was tested with positive and negative control prior to staining the samples. Antibody concentration were gathered from vendors datasheet. Isotype and tested antibody were concentration matched.
https://www.bdbiosciences.com/en-us/products/reagents/flow-cytometry-reagents/research-reagents/single-color-antibodies-ruo/apc-mouse-anti-human-hla-abc.555555
https://www.bdbiosciences.com/en-us/products/reagents/flow-cytometry-reagents/research-reagents/flow-cytometry-controls-and-lysates/apc-mouse-igg1-isotype-control.554681
https://www.bdbiosciences.com/en-us/products/reagents/flow-cytometry-reagents/research-reagents/single-color-antibodies-ruo/alexa-fluor-647-mouse-anti-human-hla-dr-dp-dq.563591
https://www.bdbiosciences.com/en-us/products/reagents/flow-cytometry-reagents/research-reagents/flow-cytometry-controls-and-lysates/alexa-fluor-647-mouse-igg2ahttps://www.biolegend.com/en-us/products/fitc-anti-human-cd39-antibody-4363https://www.biolegend.com/en-us/products/brilliant-violet-605-mouse-igg2a-kappa-isotype-ctrl-8556https://www.biolegend.com/en-us/products/brilliant-violet-605-mouse-igg2a-kappa-isotype-ctrl-8556
-isotype-control.565357
https://www.biolegend.com/it-it/products/fitc-anti-human-cd47-antibody-3707?GroupID=BLG5112
https://www.bdbiosciences.com/en-us/products/reagents/flow-cytometry-reagents/research-reagents/flow-cytometry-controls-and-lysates/fitc-mouse-igg1-isotype-control.550616
https://www.acrobiosystems.com/P5340-PE-Labeled-Monoclonal-Anti-FMC63-Antibody-Mouse-IgG1-%28Y45%29-%28Site-specific-conjugation%29-%28003%25-Proclin%29-DMF-Filed.html
https://www.biolegend.com/en-us/products/pe-mouse-igg1-kappa-isotype-ctrl-icfc-3032
https://www.biolegend.com/en-gb/search-results/brilliant-violet-605-anti-human-cd3-antibody-10421
https://www.biolegend.com/ja-jp/products/brilliant-violet-605-mouse-igg1-kappa-isotype-ctrl-7630?GroupID=ImportedGROUP1
https://www.biolegend.com/en-us/search-results/brilliant-violet-421-anti-human-cd4-antibody-7775
https://www.biolegend.com/en-us/products/brilliant-violet-421-mouse-igg2b-kappa-isotype-ctrl-7195
https://www.biolegend.com/en-us/products/percp-cyanine5-5-anti-human-cd8a-antibody-5615?GroupID=BLG5903
https://www.biolegend.com/nl-nl/products/percp-cyanine5-5-mouse-igg1-kappa-isotype-ctrl-4205
https://www.biolegend.com/en-us/products/apc-anti-human-cd19-antibody-715?GroupID=BLG10095

https://www.biolegend.com/en-us/search-results/apc-mouse-igg1-kappa-isotype-ctrl-1404
https://www.biolegend.com/en-us/products/brilliant-violet-750-anti-human-cd366-tim-3-antibody-20134
https://www.biolegend.com/en-us/products/brilliant-violet-750-mouse-igg1-%ce%ba-isotype-ctrl-15789
https://www.biolegend.com/en-us/products/apc-anti-human-tigit-vstm3-antibody-13758
https://www.biolegend.com/en-us/products/apc-mouse-igg2a-kappa-isotype-ctrl-fc-3044
https://www.biolegend.com/en-us/products/apc-anti-human-cd223-antibody-15464
https://www.biolegend.com/en-us/products/fitc-anti-human-cd39-antibody-4363
https://www.biolegend.com/en-us/products/brilliant-violet-605-anti-human-cd152-ctla-4-antibody-13910
https://www.biolegend.com/en-us/products/brilliant-violet-605-mouse-igg2a-kappa-isotype-ctrl-8556
https://www.biolegend.com/en-us/products/fitc-anti-human-cd279-pd-1-antibody-21956
https://www.biolegend.com/en-us/products/fitc-mouse-igg2b-kappa-isotype-ctrl-1412

Eukaryotic cell lines

Policy information about cell lines and Sex and Gender in Research

Cell line source(s)	Nalm6 fluc+ cells were purchased from Creative Biogene (cat.no. CSC-RR0361. Daudi cells were purchased from ATCC (cat.no. cat.no. CCL-213). HEK293LX were purchased fromTakara (cat.no. 632180).
Authentication	None of the cell lines used have been authenticated.
Mycoplasma contamination	All cell lines were tested and negative for mycoplasma contamination using the Universal Mycoplasma test kit from ATCC.
Commonly misidentified lines (See ICLAC register)	No commonly misidentified cell lines were used

Animals and other research organisms

Policy information about studies involving animals; ARRIVE guidelines recommended for reporting animal research, and Sex and Gender in Research

Laboratory animals	Male and female NSG mice (005557) and female humanized NSG-SGM3 mice (013062) were purchased from the Jackson Laboratories and served as recipients for different assays. Humanized mice were not thymectomized, received human CD34+ cells at 12 weeks of age, and were included into study groups 6 – 8 weeks after humanization. Mice were housed in 12 hour light-dark cycles with humidity between 30-70% at ambient temperature of 20-26 degrees Celsius. The study and control animals were housed in the same room. The animal facility is a specific pathogen-free facility. Euthanasia was conducted via exsanguination under isoflurane anesthesia followed by cervical dislocation.
Wild animals	No wild animals were used
Reporting on sex	study design was based on male and female NSG mice overall. Humanized mice are only available as females.
Field-collected samples	No field collected samples were used
Ethics oversight	Mice received humane care in compliance with the IACUC and performed according to California's guidelines.

Note that full information on the approval of the study protocol must also be provided in the manuscript.

Flow Cytometry

Plots

Confirm that:

- [x] The axis labels state the marker and fluorochrome used (e.g. CD4-FITC).
- [x] The axis scales are clearly visible. Include numbers along axes only for bottom left plot of group (a 'group' is an analysis of identical markers).
- [x] All plots are contour plots with outliers or pseudocolor plots.
- [x] A numerical value for number of cells or percentage (with statistics) is provided.

Methodology

Sample preparation	Unmodified and modified T cells were counted, stained and measured as single cell suspension in PBS+2% FCS hi. Single cells were generated from mice bone marrow and spleen. Cells were counted and stained in PBS+2% FCS hi.
Instrument	BD Aria Fusion (BD Bioscience) or Cytoflex (Beckman Counter) were used.
Software	The FACS Diva 9.0 software , CytExpert 2.4 software or Atune NxT 5.1 software were used.

Cell population abundance	For flow cytometry analysis, more than 10,000 positive cells were measured. Cell sorting was gated for the desired population and sorted for the cell amount needed for assays.
Gating strategy	Samples were gated in FSC/SSC for the correct cell size and live cells. Isotype was measured for each sample as defined as unspecific staining threshold.

☒ Tick this box to confirm that a figure exemplifying the gating strategy is provided in the Supplementary Information.
